# Hyperoside mitigates photoreceptor degeneration in part by targeting cGAS and suppressing DNA-induced microglial activation

**DOI:** 10.1186/s40478-024-01793-0

**Published:** 2024-05-16

**Authors:** Daijin Li, Jie Chang, Yujue Wang, Xiaoye Du, Jing Xu, Jingang Cui, Teng Zhang, Yu Chen

**Affiliations:** 1 Yueyang Hospital of Integrated Traditional Chinese and Western Medicine, Shanghai University of Traditional Chinese Medicine, Shanghai, 200437 China; 2 Clinical Research Institute of Integrative Medicine, Shanghai Academy of Traditional Chinese Medicine, Shanghai, 200437 China; 3 Laboratory of Clinical and Molecular Pharmacology, Yueyang Hospital of Integrated Traditional Chinese and Western Medicine, Shanghai University of Traditional Chinese Medicine, Shanghai, 200437 China

**Keywords:** Hyperoside, Photoreceptor degeneration, Neuroinflammation, Microglia activation, cGAS

## Abstract

Activated microglia play an important role in driving photoreceptor degeneration-associated neuroinflammation in the retina. Controlling pro-inflammatory activation of microglia holds promise for mitigating the progression of photoreceptor degeneration. Our previous study has demonstrated that pre-light damage treatment of hyperoside, a naturally occurring flavonol glycoside with antioxidant and anti-inflammatory activities, prevents photooxidative stress-induced photoreceptor degeneration and neuroinflammatory responses in the retina. However, the direct impact of hyperoside on microglia-mediated neuroinflammation during photoreceptor degeneration remains unknown. Upon verifying the anti-inflammatory effects of hyperoside in LPS-stimulated BV-2 cells, our results here further demonstrated that post-light damage hyperoside treatment mitigated the loss of photoreceptors and attenuated the functional decline of the retina. Meanwhile, post-light damage hyperoside treatment lowered neuroinflammatory responses and dampened microglial activation in the illuminated retinas. With respect to microglial activation, hyperoside mitigated the pro-inflammatory responses in DNA-stimulated BV-2 cells and lowered DNA-stimulated production of 2′3′-cGAMP in BV-2 cells. Moreover, hyperoside was shown to directly interact with cGAS and suppress the enzymatic activity of cGAS in a cell-free system. In conclusion, the current study suggests for the first time that the DNA sensor cGAS is a direct target of hyperoside. Hyperoside is effective at mitigating DNA-stimulated cGAS-mediated pro-inflammatory activation of microglia, which likely contributes to the therapeutic effects of hyperoside at curtailing neuroinflammation and alleviating neuroinflammation-instigated photoreceptor degeneration.

**Supplementary Information:**

The online version contains supplementary material available at 10.1186/s40478-024-01793-0.

## Introduction

Photoreceptors are the first-order retinal neurons that carry out light-sensing and phototransduction functions indispensable for vision formation [1]. Photoreceptor degeneration directly leads to progressive vision impairment in patients with age-related macular degeneration and retinitis pigmentosa or in patients with systemic disorders that affect the photoreceptors in a secondary manner [2–4]. Primary or secondary in nature, photoreceptor degeneration remains currently untreatable presumably due to complexity of the disease mechanisms. Accumulating evidence has indicated that the neuroinflammatory responses in the retina arise as a common feature shared by photoreceptor degenerative diseases of different etiologies. Microglia, the resident macrophages activated following photoreceptor damage, play an important part in orchestrating neuroinflammation that further exacerbates photoreceptor loss. Thus, it has been proposed that aberrant microglial activation could be targeted to dampen neuroinflammation, thereby alleviating the loss of photoreceptors and improving the outcome of photoreceptor degenerative diseases [5, 6].

Hyperoside (quercetin-3-O-β-D-galactoside) is a naturally occurring flavonol glycoside equipped with antioxidant and anti-inflammatory activities [7]. It has been shown that hyperoside suppresses the pro-inflammatory activation of microglia and attenuates the loss of dopaminergic neurons in mice recapitulating Parkinson’s disease [8, 9], supporting the therapeutic potentials of hyperoside in alleviating neuroinflammation in neurodegenerative disorders. Moreover, our previous work has demonstrated that hyperoside prevents photooxidative stress-induced photoreceptor degeneration, neuroinflammatory responses and microglial activation in vivo [10]. However, in our previous work, in order to assess the photoreceptor protective effects of hyperoside as an antioxidant, instead of post-light damage treatment, one-dose pre-light damage hyperoside treatment was administered to counteract photooxidative stress that triggers the initial loss of photoreceptors. Given that microglial activation occurs as a result of stressed or damaged photoreceptors, our one-dose pretreatment regimen is not sufficient to justify the therapeutic potentials of hyperoside, as an anti-inflammatory agent, in controlling the further loss of photoreceptors promoted by activated microglia-mediated neuroinflammatory responses. Thus, the pharmacological implications of hyperoside in controlling microglial activation in the pathological context of photoreceptor degeneration remain to be investigated.

Therefore, the current study set out to validate the impact of hyperoside on the pro-inflammatory activation of microglia, followed by assessing its effects at mitigating neuroinflammation-instigated photoreceptor degeneration. Lastly, the mechanisms of hyperoside on microglial activation were further probed.

## Materials and methods

### Reagents

Hyperoside (purity ≥ 98%, Lot. No. JOT-10,193) was ordered from Chengdu Pufei De Biotech Co., Ltd (China). 2-Aminoethyl diphenylborinate (2-APB) and lipopolysaccharides (LPS) were obtained from Sigma-Aldrich (USA). Calf thymus DNA (ctDNA), Lipofectamine 2000, Dulbecco’s Modified Eagle’s Medium (DMEM), and penicillin/streptomycin were products from Thermo Fisher Scientific (USA). Fetal bovine serum (FBS) was ordered from Nobimpex (Germany). 2′,3′-Cyclic guanosine monophosphate–adenosine monophosphate (cGAMP) sodium and 5,6-dimethyl-9-oxo-9 H-xanthene-4-acetic acid (DMXAA) were products from MedChemExpress (USA). Evans blue dye was purchased from Solarbio (China).

### Cell culture

The BV-2 microglial cell line, acquired from the Cell Bank of the Chinese Academy of Sciences (China), was cultured using DMEM supplemented with 10% FBS and 1% penicillin/streptomycin at 37 °C in an atmosphere of 5% CO_2_. For LPS stimulation experiments, BV-2 cells were treated with hyperoside at the specified doses or vehicle for 1 h, followed by stimulation with LPS at 20 ng/ml for 12–24 h. For experiments involving ctDNA, after treating BV-2 cells with hyperoside at the specified doses or vehicle for 1 h, the cells were stimulated with ctDNA at 1 µg/ml for 3–6 h. For experiments involving DMXAA and 2′3′-cGAMP, BV-2 cells were treated with hyperoside or a vehicle for 1 h, followed by DMXAA stimulation at 10 µg/ml or 2′3′-cGAMP stimulation at 20 µM for 3–6 h. Transfection of ctDNA or 2′3′-cGAMP was performed using Lipofectamine 2000 following the manufacturer’s instructions. The doses and time points for the indicated treatments were determined by preliminary experiments.

### Enzyme-linked immunosorbent assay (ELISA)

The supernatants from the BV-2 cells were collected after the indicated treatments. The levels of tumor necrosis factor (TNF), interleukin 6 (IL6), chemokine ligand 2 (CCL2) and interferon beta (IFNB) were then quantified using ELISA. Briefly, BV-2 cells were seeded at 6 × 10^4^ cells per well in 24-well plates and stimulated with LPS, ctDNA, DMXAA or 2′3′-cGAMP in the absence or presence of hyperoside. The supernatants were then collected and subjected to the measurement using the Mouse TNF alpha Uncoated ELISA Kit (Thermo Fisher Scientific, USA), Mouse IL-6 Uncoated ELISA Kit (Thermo Fisher Scientific, USA), Mouse MCP-1/CCL2 Uncoated ELISA Kit (Thermo Fisher Scientific, USA), or Mouse IFN beta ELISA Kit (Multi Sciences, China) following the manufacturers’ instructions. For measuring the amount of 2′3′-cGAMP, BV-2 cells were lysed in RIPA buffer (Beyotime, China) containing proteinase inhibitors (Roche, Germany) on ice for 30 min, followed by centrifugation at 12,000 × g at 4°C for 30 min. The supernatant was then collected and analyzed using the 2′3′-cGAMP ELISA Kit (Cayman Chemical, USA) following the manufacturer’s instructions. For measuring the amount of 2′3′-cGAMP in cell-free system, hyperoside or CU-76, a cyclic GMP-AMP synthase (cGAS) inhibitor serving as the positive control, was co-incubated with cGAS in the presence of DNA, ATP, and GTP for 30 min, followed by measuring the amount of 2′3′-cGAMP using the cGAS Inhibitor Screening Assay Kit (Cayman Chemical, USA) according to the manufacturer’s instructions.

### Scratch wound assay

The migratory capacity of BV-2 cells was evaluated by a scratch wound assay using the IncuCyte system (Essen Biosciences, German). In brief, BV-2 cells were seeded at 3 × 10^4^ per well on 96-well ImageLock plates and were allowed to settle for 16 h. Subsequently, a scratch wound was created in each well using a 96-pin WoundMaker. The wells were then washed once with DMEM before adding fresh media containing either vehicle, LPS or hyperoside at the indicated concentrations. Images were automatically captured at the 1-h interval and recorded for the duration of 24 h using the IncuCyte Zoom with a 10× objective (Essen Biosciences, German). The data were then analyzed using an integrated metric of Incucyte, relative wound density. This algorithm measures cell density within the wound area relative to the cell density outside of the wound area at the specified time point. At 0 h post wounding, the relative wound density is set to be 0. When the cell density inside of the wound is the same as the cell density outside of the initial wound, the relative wound density is 100%.

### Animals and treatments

Six to twelve-week-old female Balb/c mice were obtained from the Shanghai Laboratory Animal Research Center and maintained under the laboratory conditions including a 12/12-h light-dark cycle and the room temperature set at 20 ± 2 °C. Dark-adapted mice were subjected to 30-min experimental light exposure (10,000 lx) (Compact Fluorescence Lamp, 45 W, Chaoya Lighting, Shanghai, China), followed by intraperitoneal injection of hyperoside (prepared in 0.5% sodium carboxymethyl cellulose solution) at 100 mg/kg twice a day for 7 d. The first dose of hyperoside was delivered 3 h after the illumination. The volume for intraperitoneal injection of hyperoside was set at 100 µl per mouse. 2-APB (dissolved in DMSO) was administered intraperitoneally at 2.5 or 10 mg/kg 30 min before illumination for one-dose pretreatment and at 10 mg/kg twice a day for post-light damage treatment. The volume for 2-APB treatment was controlled at 50 µl per mouse. Normal controls (the mice unexposed to the experimental light) and the light-exposed mice without hyperoside or 2-APB treatment received vehicle treatment in the same manner. The animal handling protocols were reviewed and approved by the Institutional Animal Care and Use Committee at Yueyang Hospital of Integrated Traditional Chinese and Western Medicine, Shanghai University of Traditional Chinese Medicine (YYLAC-2023-199-1). The experiments were conducted in accordance with the NIH Guide for the Care and Use of Laboratory Animals and the ARVO Statement for the Use of Animals in Ophthalmic and Vision Research.

### Evans blue dye uptake assay

Evans blue dye was injected via tail vein at 25 mg/kg. The mice were then subjected to illumination at 10,000 lx for 30 min. The eyes were enucleated at 1, 3 and 6 h post illumination. The eye cups free of the cornea and lens were then made and processed for cryosectioning. The autofluorescence of Evans blue dye was recorded using a fluorescent microscope (DM6000B, Leica, Germany). Quantification of the area of Evans blue autofluorescence in the outer nuclear layer (ONL) was performed using ImageJ.

### Optical coherence tomography (OCT)

Image-guided OCT (OCT 2 with Micron IV, Phoenix Research Labs, USA) was performed to scan the retina 7 d post illumination as previously described [10]. In brief, after administering anesthetic cocktail of ketamine hydrochloride (82.5 mg/kg body weight) and xylazine (8.25 mg/kg body weight), pupil dilation was induced using 1% tropicamide (Santen Pharmaceutical, Japan) to prepare the mice for the OCT imaging. Five full-retinal scans were obtained and automatically averaged using the Phoenix Reveal OCT software (Phoenix Research Labs, USA). The thickness of the ONL was measured using the Insight Image Segmentation Software, an adjunct software to the Phoenix OCT and Retinal Imaging System (Version 2.0.5490, Voxeleron LLC, USA).

### Electroretinography (ERG)

Seven days after the illumination, dark-adapted mice were subjected to ERG analysis under a safe light condition (5 lx) as previously described [10]. Flashes of green light (504 nm) were delivered at intensities of -2 (0.5 msec duration and 5 s inter-stimulus-interval), -0.8 (1 msec duration and 5 s inter-stimulus-interval), 0.4 (1 msec duration and 10 s inter-stimulus-interval), 1.6 (1 msec duration and 20 s inter-stimulus-interval), and 3.1 (1 msec duration and 60 s inter-stimulus-interval) log cd·s·m^− 2^. ERG responses were recorded using the Ganzfeld ERG system and analyzed by LabScribe software (ERG 2, Phoenix Research Labs, USA).

### Histological examination and immunohistochemistry (IHC)

The enucleated eyes were fixed in 4% paraformaldehyde for 24 h prior to further processing for paraffin sectioning. Paraffin Sect. (4-µm-thick) were stained with hematoxylin and eosin (HE) or subjected to IHC examination using the primary antibodies including mouse anti-double stranded DNA (dsDNA) (1:500) (Santa Cruz, USA) and rabbit anti-high mobility group box 1 (HMGB1) (1:500) (Millipore, USA) as well as the secondary antibodies including Cy3-conjugated sheep anti-mouse (1:1000) or Cy3-sheep anti-rabbit secondary antibodies (1:1000) (Sigma-Aldrich, USA). Additionally, eye cups were fixed in 4% paraformaldehyde for 2 h at room temperature and processed for cryosectioning. Cryosections (12-µm-thick) were examined by IHC using primary antibodies including rabbit anti-ionized calcium binding adaptor molecule 1 (Iba-1) (1:500) (Wako Chemicals, USA), rabbit anti-CD68 (1:500) (Abcam, USA), rabbit anti-phospho-stimulator of interferon genes (STING) (Ser366) (1:250) (Thermo Fisher, USA), rat anti-F4/80 (1:250) (Thermo Fisher, USA), and the secondary antibodies including Cy3-conjugated sheep anti-rabbit (1:1000) (Sigma-Aldrich, USA), Cy3-conjugated goat anti-rat (1:1000) (Abcam, USA) or FITC-conjugated donkey anti-rabbit (1:1000) (Sigma-Aldrich, USA). Nuclei counterstaining was performed with 4-6-diamidino-2-phenylindole (DAPI) (Sigma Aldrich, USA). Microscopic imaging was conducted using a light microscope (DM2000, Leica, Germany) or a fluorescent microscope (DM6000B, Leica, Germany). Quantification of dsDNA positivity in the ONL was performed using ImageJ.

### Real-time quantitative PCR (qPCR)

Total RNA from the mouse retinas was isolated using TRIzol reagent (Invitrogen, USA). Reverse transcription was then performed using PrimeScript RT Master Mix (TaKaRa, Japan). SYBR Green I Master (Roche, USA) was used to set up PCR reactions, which were run on a Roche Light Cycler 480 II (Roche, USA). The expression of *Ccl5*, *Ccl6*, *Cd68*, *Cgas*, *Cxcl10*, *Ifi202b*, *Ifnb*, *Ikbke*, *Il6*, *Irf7*, *Sting1* and *Tnf* was analyzed along with the internal control *18 S rRNA*. The primer sequences are included in Table 1. The fold change in gene expression was calculated based on 2^−[Ct (candidate gene)−Ct (18s rRNA)]^.

**Table 1:** Primer sequences

Gene name	Forward primer (5’-3’)	Reverse primer (5’-3’)
*Ccl5*	CCTGCTGCTTTGCCTACCTCTC	ACACACTTGGCGGTTCCTTCGA
*Ccl6*	TGCCACACAGATCCCATGTA	TCCTGCTGATAAAGATGATGCC
*Cd68*	GGCGGTGGAATACAATGTGTCC	AGCAGGTCAAGGTGAACAGCTG
*Cgas*	GAGGCGCGGAAAGTCGTAA	TTGTCCGGTTCCTTCCTGGA
*Cxcl10*	ATCATCCCTGCGAGCCTATCCT	GACCTTTTTTGGCTAAACGCTTTC
*Ifi202b*	GACCCCTTCCAGTGATTCATCT	ACAGCACCTTTGCTAATGTTCT
*Ifnb*	CAGCTCCAAGAAAGGACGAAC	GGCAGTGTAACTCTTCTGCAT
*Ikbke*	ACCACTAACTACCTGTGGCAT	CCTCCCCGGATTTCTTGTTTC
*Il6*	GCCTTCTTGGGACTGATGCT	TGCCATTGCACAACTCTTTTCT
*Irf7*	GAGACTGGCTATTGGGGGAG	GACCGAAATGCTTCCAGGG
*Sting1*	GGTCACCGCTCCAAATATGTAG	CAGTAGTCCAAGTTCGTGCGA
*Tnf*	ACGTCGTAGCAAACCACCAA	GCAGCCTTGTCCCTTGAAGA
*18 S rRNA*	GAGGTTCGAAGACGATCAGA	TCGCTCCACCAACTAAGAAC

### Western blotting

Cell lysates were extracted from BV-2 cells using RIPA lysis buffer (Beyotime, China) supplemented with protease and phosphatase inhibitors (Roche, Germany). The lysates were then run on 10% SDS-PAGE gels and transferred onto polyvinylidene fluoride membranes (Millipore, USA). Primary antibodies used for Western blotting included monoclonal rabbit anti-cGAS (#31659S, Cell Signaling Technology, USA), monoclonal rabbit anti-phospho-tank binding kinase 1 (TBK1)/NAK (Ser172) (#5483S, Cell Signaling Technology, USA) and monoclonal rabbit anti-TBK1/NAK (#ab40676, Abcam, USA). Following incubation with the primary antibodies, horseradish peroxidase-conjugated goat anti-rabbit secondary antibody (Promega, USA) and the WesternBright ECL reagent (Advansta, USA) were sequentially applied. The blots were scanned using a ChemiScope 6000 imaging system (Clinx Science Instruments, China). Densitometry analysis was conducted using ImageJ.

### Molecular docking and molecular dynamics (MD) simulations

The crystal structure of mouse cGAS was obtained from the Protein Data Bank (PDB ID: 4O6A) and the molecular structure of hyperoside was downloaded from PubChem (Compound CID: 5,281,643). Prior to the docking analysis, water molecules and original cGAS ligands such as DNA were removed from the file of cGAS. The polar hydrogen atoms and Gasteiger charges were added to hyperoside and cGAS, respectively. Non-polar hydrogen atoms were merged using AutoDock Tools1.5.6 software. A semi-flexible docking process between hyperoside and cGAS was performed using Autodock Vina software. The specific parameter is center_x=-31.129, center_y=-18.476 and center_z = 36.01. A total of 100 docking conformations were generated and ranked based on their docking energy values. MD simulation was then conducted using the GROMACS 19.5 package (https://manual.gromacs.org/) with the TIP3P explicit water model. The system was electrically neutralized by adding counterions. Subsequently, energy minimization (1000.0 kJ/mol/nm) was performed using the steepest descent method. Canonical ensemble simulation (NVT, 400 ps) and isothermal isobaric simulation (NPT, 400 ps) were used to guarantee that the system could endure constant temperature and pressure (300 K, 1.01325 bar). The annealing method was used to make sure that the system was slowly warmed from 0 to 300 K within 150 ps in the NVT simulation process. Lastly, a 50-ns MD simulation was started. The most populated cluster during the 30–50 ns MD simulation was selected for detailed analysis of the binding modes.

### Surface plasmon resonance (SPR)

SPR was performed at 25 °C in the LMW multi-cycle mode on a BIAcore T200 instrument (Cytiva, USA). Briefly, the NTA biosensor chip (Cytiva, USA) was immobilized with human recombinant cGAS. Hyperoside were then flowed over the chip at the indicated concentrations (1.56 µM, 3.125 µM, 6.25 µM, 12.5 µM, 25 µM, 50 µM, 100 µM, and 200 µM) and the real-time responses were recorded. The binding affinity was assessed based on the equilibrium dissociation constant (K_D_) calculated using the BIAcore T200 evaluation software (Cytiva, USA).

### Cellular thermal shift assay (CETSA)

BV-2 cells were treated with 200 µM hyperoside or DMSO for 3 h. The cells were then washed 3 times with PBS and resuspended in 1 ml PBS containing protease inhibitors (Roche, Germany). The cell suspensions were heated at 48 °C, 51 °C, 54 °C, 57 °C, and 60 °C. The cells were then processed for 3 cycles of snap-freezing in liquid nitrogen and thawing at 25 °C. Centrifugation was subsequently performed at 12,000 × g at 4 °C for 30 min to collect the supernatants. Equal amount of the supernatant from each treatment group was then subjected to Western blotting to assess the protein level of cGAS. The level of β-actin was simultaneously probed as a loading control. The protein bands were visualized using NBT/BCIP substrates (Solarbio, China).

### Statistical analysis

Data were presented as mean ± standard error of the mean (SEM). Statistical analyses were conducted using one-way or two-way ANOVA, followed by Tukey’s multiple comparisons test (GraphPad Prism 9, USA). A P value less than 0.05 was considered statistically significant.

## Results

### Hyperoside suppresses LPS-stimulated pro-inflammatory activation of microglia

**Fig. 1:**
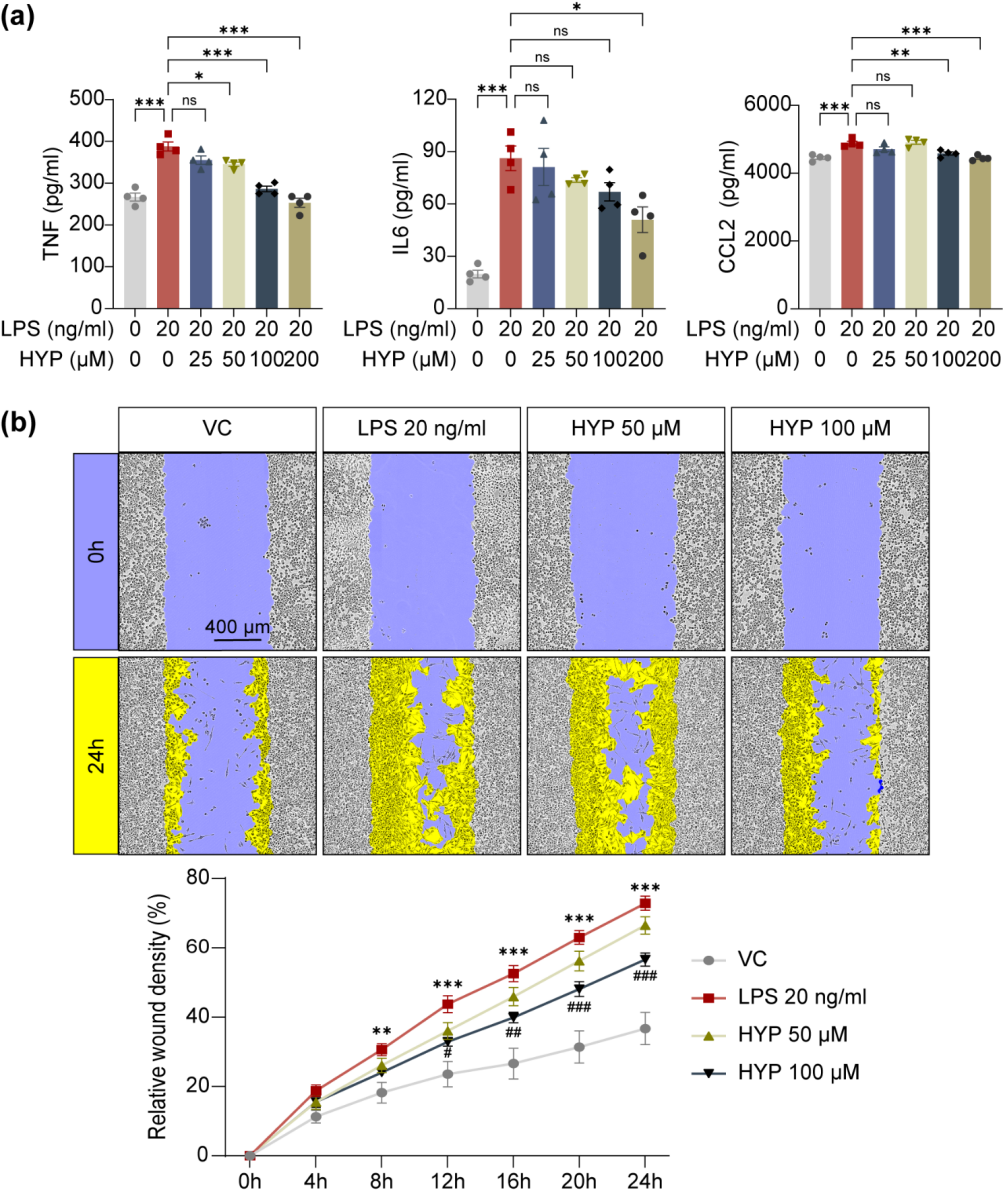
Hyperoside suppresses LPS-induced pro-inflammatory responses in BV-2 cells. **(a)** The cell culture supernatants were collected 12 h after LPS stimulation, followed by ELISA to quantify the amount of TNF, IL6 and CCL2. **(b)** The scratch-wound healing assay was performed by measuring the wound closure in BV-2 cells up to 24 h at the 1-h interval. Representative micrographs from the indicated treatments were shown in the top panel. Relative wound density was plotted and presented in the bottom panel. Scale bar, 400 μm. Data were expressed as mean ± SEM (*n* = 4–6 per group). ^**^Compared to VC, *P* < 0.01; ^***^compared to VC, *P* < 0.001; ^#^ compared to LPS, *P* < 0.05; ^##^ compared to LPS, *P* < 0.01; ^###^ compared to LPS, *P* < 0.001. ns, not significant. VC, vehicle-treated cells without LPS stimulation; LPS, vehicle-treated LPS-stimulated cells; HYP, LPS-stimulated cells treated with hyperoside at the indicated doses

Prior to exploring the therapeutic implications of hyperoside in attenuating photoreceptor degeneration-associated microglia activation and neuroinflammation in vivo, the direct impact of hyperoside on microglial activation was validated by measuring LPS-induced production of pro-inflammatory TNF, IL6 and CCL2 in BV-2 cells. As shown in Fig. 1a, in contrast to augmented production of TNF, IL6 and CCL2 in the vehicle-treated LPS-stimulated cells, lower levels of these pro-inflammatory factors were noted in the LPS-stimulated cells treated with hyperoside at the indicated doses. Furthermore, the impact of hyperoside on LPS-stimulated migration of BV-2 cells was monitored by automated continuous live cell imaging analysis. As expected, LPS stimulation enhanced the migration of BV-2 cells, whereas hyperoside dose-dependently attenuated LPS-stimulated migration of BV-2 cells (Fig. 1b). These results confirm that hyperoside is effective at mitigating the pro-inflammatory activation of microglia.

### Post-light damage treatment of hyperoside partially preserves the retinal structure, function and neuroimmune homeostasis

**Fig. 2:**
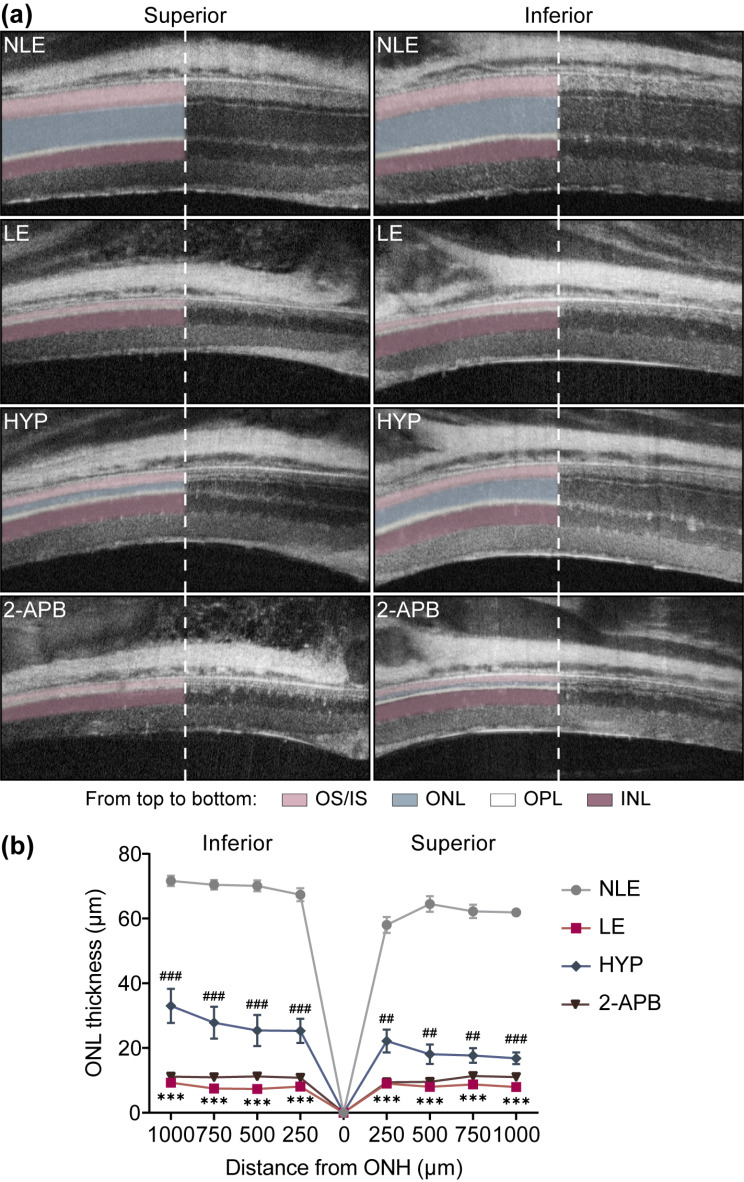
Post-light damage treatment of hyperoside alleviates photoreceptor structural impairment. Hyperoside (100 mg/kg) or 2-APB (10 mg/kg) was administered starting from 3 h post illumination and carried out twice a day for 7 d. **(a)** Representative OCT scans of the superior and inferior retinas taken from day 7 post light exposure. **(b)** The ONL thickness measured at 250, 500, 750, and 1000 μm away from the ONH. Data were expressed as mean ± SEM (*n* = 6 per group). ^***^Compared to NLE, *P* < 0.001; ^##^compared to LE, *P* < 0.01, ^###^compared to LE, *P* < 0.001. NLE, the vehicle-treated mice without light exposure; LE, the light-exposed mice treated with vehicle; HYP, the hyperoside-treated light-exposed mice; 2-APB, the 2-APB-treated light-exposed mice; INL, inner nuclear layer; IS, inner segment; ONH, optic nerve head; ONL, outer nuclear layer; OPL, outer plexiform layer; OS, outer segment

**Fig. 3:**
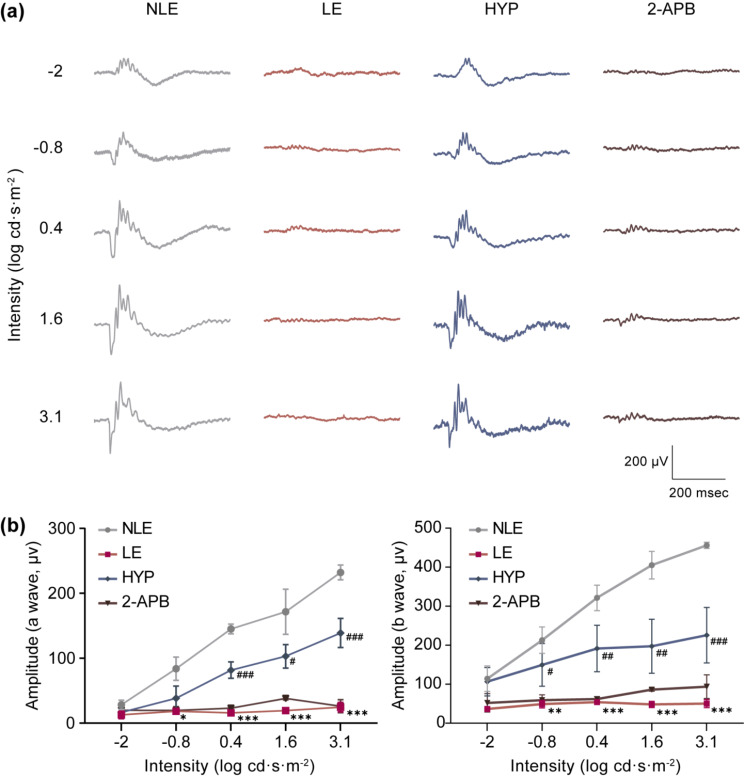
Post-light damage treatment of hyperoside improves the retinal function. Hyperoside (100 mg/kg) or 2-APB (10 mg/kg) was administered starting from 3 h post illumination and carried out twice a day for 7 d. ERG was recorded after the indicated treatments. **(a)** Representative scotopic ERG waves. **(b)** Amplitudes of a wave and b wave were plotted. Data were presented as mean ± SEM (*n* = 6 per group). ^*^Compared to NLE, *P* < 0.05; ^**^compared to NLE, *P* < 0.01; ^***^compared to NLE, *P* < 0.001; ^#^compared to LE, *P* < 0.05; ^##^compared to LE, *P* < 0.01; ^###^compared to LE, *P* < 0.001. NLE, the vehicle-treated mice without the experimental light exposure; LE, the vehicle-treated light-exposed mice; HYP, the vehicle-treated light-exposed mice; 2-APB, the 2-APB-treated light-exposed mice

**Fig. 4:**
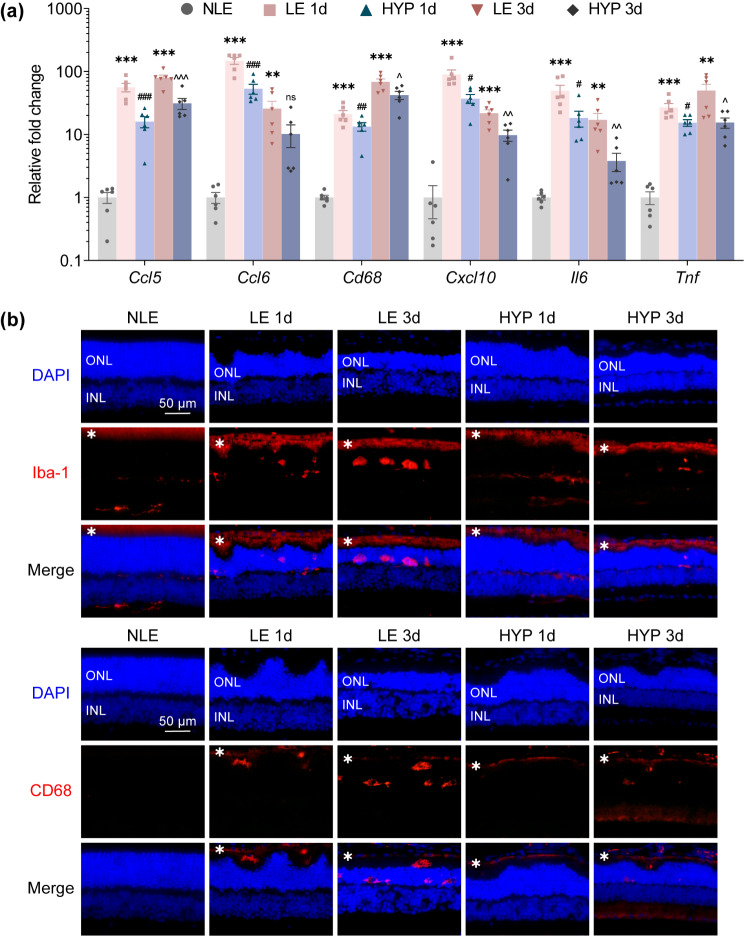
Post-light damage treatment of hyperoside mitigates the inflammatory responses and microglial activation in the retina. Hyperoside was administered at 100 mg/kg starting from 3 h post illumination and carried out twice a day for 1 d or 3 d. **(a)** Real-time qPCR was performed to analyze the expression of *Ccl5*, *Ccl6*, *Cd68*, *Cxcl10*, *Il6* and *Tnf* in the retinas collected 1 d or 3 d after the indicated treatments. Relative fold change was plotted against that NLE (y-axis, log10 scale). **(b)** IHC was performed to assess the immunopositivity of Iba-1 (in red) and CD68 (in red) in the retina. Nuclei were counterstained by DAPI (in blue). White asterisks indicate nonspecific background. Scale bar, 50 μm. Data were presented as mean ± SEM (*n* = 6 per group). ^**^Compared to NLE, *P* < 0.01; ^***^compared to NLE, *P* < 0.001; ^#^compared to LE 1d, *P* < 0.05; ^##^compared to LE 1d, *P* < 0.01; ^###^compared to LE 1d, *P* < 0.001. ^^^compared to LE 3d, *P* < 0.05; ^^^^compared to LE 3d, *P* < 0.01; ^^^^^compared to LE 3d, *P* < 0.001; ns, not significant. NLE, the vehicle-treated mice without the experimental light exposure; LE, the vehicle-treated light-exposed mice; HYP, the vehicle-treated light-exposed mice; INL, inner nuclear layer; ONL, outer nuclear layer

Following the onset of photoreceptor stress or damage, microglia are activated and uncontrolled microglial activation further exacerbates photoreceptor degeneration via neurotoxic effects [6]. As shown in Fig. 1, hyperoside mitigates the pro-inflammatory activation of BV-2 cells, raising the possibility that by curtailing microglial activation, hyperoside may alleviate the progression of photoreceptor degeneration. To directly test this hypothesis, instead of delivering a one-dose pre-light damage hyperoside treatment to antagonize photooxidative stress [10], a post-light damage hyperoside treatment regimen was adopted. Release of non-histone nuclear protein HMGB1 signifies necrotic cell damage and ensuing inflammation [11]. This phenomenon has been noted in the light-damaged retinas [12]. Consistently, our previous study has also documented that degenerative photoreceptors (1 d and 3 d post illumination) are characterized by diminishment in the nuclear HMGB1 [13]. Thus, the loss of the nuclear signal of HMGB1 in photoreceptors could be regarded as a biomarker indicative of compromised photoreceptor integrity. To determine the appropriate time-point to start post-light damage hyperoside treatment, IHC was performed to track the changes in the nuclear signal of HMGB1 at earlier time points (1 h and 3 h) post illumination. As shown in Supplementary Fig. 1, overt diminishment of HMGB1 immunopositivity was readily observed in the ONL 1 h and 3 h post illumination. Therefore, the first dose of hyperoside was administered 3 h after the illumination and carried out for 7 d. At the end of the indicated treatments, OCT imaging revealed prominent impairment of the ONL and reductions in the ONL thickness in both superior and inferior retinas in the vehicle-treated light-exposed mice compared to the normal controls. However, in the hyperoside-treated light-exposed mice, partial preservation of the ONL structure was noted, particularly in the inferior retinas (Supplementary Fig. 2a, b). Histological examination validated the beneficial effects of hyperoside on curtailing light-induced photoreceptor loss (Supplementary Fig. 2c, d). Consistent with the structural protection, the retinal function was also partially maintained by hyperoside treatment as revealed by ERG analyses (Supplementary Fig. 3a, b). To rule out the involvement of direct photoreceptor protective activities of hyperoside in the aforementioned effects, the experiment was repeated alongside 2-APB, an inositol 1,4,5-triphosphate antagonist that blocks photooxidative stress-triggered intracellular Ca^2+^ rise in photoreceptors and prevents light-induced photoreceptor degeneration when administered prior to illumination [14]. After verifying the reported photoreceptor protection conferred by one-dose 2-APB pre-light exposure treatment (Supplementary Fig. 4), parallel post-light damage treatment of 2-APB was performed to serve as a negative control for post-light damage hyperoside treatment. Partial preservation of the ONL was only noted in the light-exposed hyperoside-treated retinas but not in the 2-APB-treated light-exposed retinas (Fig. 2a, b). Consistently, compared to the markedly impaired retinal function detected in the vehicle-treated light-exposed mice, significant increases in the a-wave and b-wave amplitudes were noted in the hyperoside-treated light-exposed mice. No improvement of the retinal function was seen in the 2-APB-treated light-exposed retinas (Fig. 3a, b). Furthermore, the retinal expression of the genes encoding pro-inflammatory factors and the markers of microglia/macrophages was analyzed. As shown in Fig. 4a, significantly elevated expression of *Ccl5, Ccl6, Cd68, Cxcl10, Il6* and *Tnf* was noted in the vehicle-treated light-exposed retinas compared to the normal controls. In contrast, much lower retinal expression of these genes was observed in the hyperoside-treated light-exposed mice compared to vehicle-treated light-exposed mice. Consistently, the presence of Iba-1 and CD68 positive microglia in the outer retinas in the light-exposed mice was significantly attenuated as a result of post-light damage hyperoside treatment (Fig. 4b). Collectively, these results demonstrate that post-light damage hyperoside treatment is effective at attenuating photoreceptor degeneration and microglial activation-associated neuroinflammation.

### Hyperoside mitigates photoreceptor degeneration-associated upregulation of cytosolic DNA-sensing pathway in the retina

**Fig. 5:**
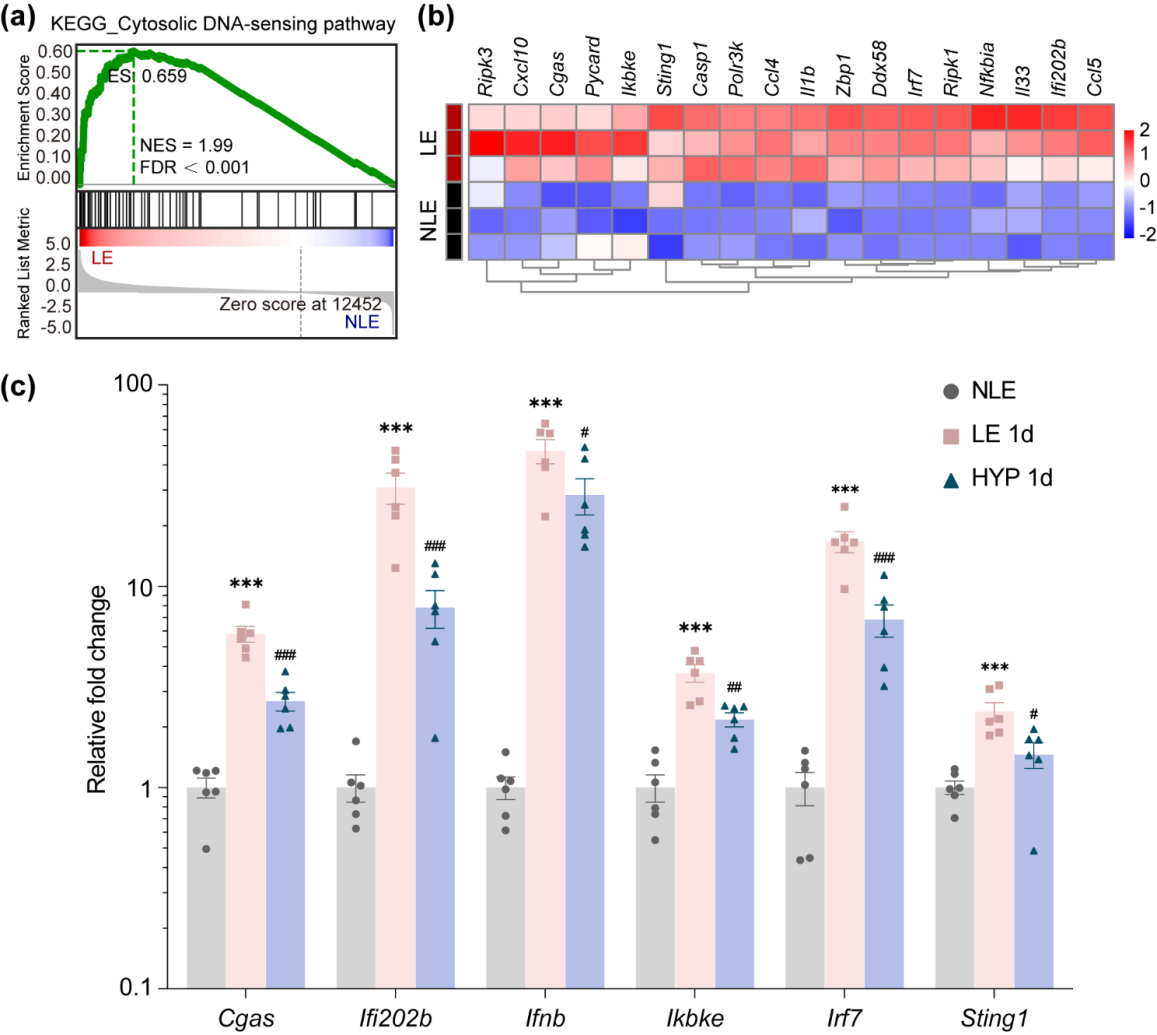
Post-light damage treatment of hyperoside lowers the retinal expression of genes involved in cytosolic DNA-sensing pathway. **(a)** Gene set enrichment analysis was derived from our previously published RNA-sequencing data [10]. **(b)** Heatmap visualization of the representative genes identified in cytosolic DNA-sensing pathway [10]. **(c)** Hyperoside was administered to the light-exposed mice 3 h after illumination and the retinas were isolated 24 h later. The retinal expression of the indicated genes was analyzed by real-time qPCR. Relative fold change was normalized against NLE (y-axis, log10 scale). Data were expressed as mean ± SEM (*n* = 6 per group). ^**^Compared to NLE, *P* < 0.01; ^***^compared to NLE, *P* < 0.001; ^#^compared to LE, *P* < 0.05; ^##^compared to LE, *P* < 0.01; ^###^compared to LE, *P* < 0.001; ns, not significant. NLE, the vehicle-treated mice without the experimental light exposure; LE, the vehicle-treated light-exposed mice; HYP, the hyperoside-treated light-exposed mice; ES, enrichment score; NES, normalized enrichment score; FDR, false discovery rate

**Fig. 6:**
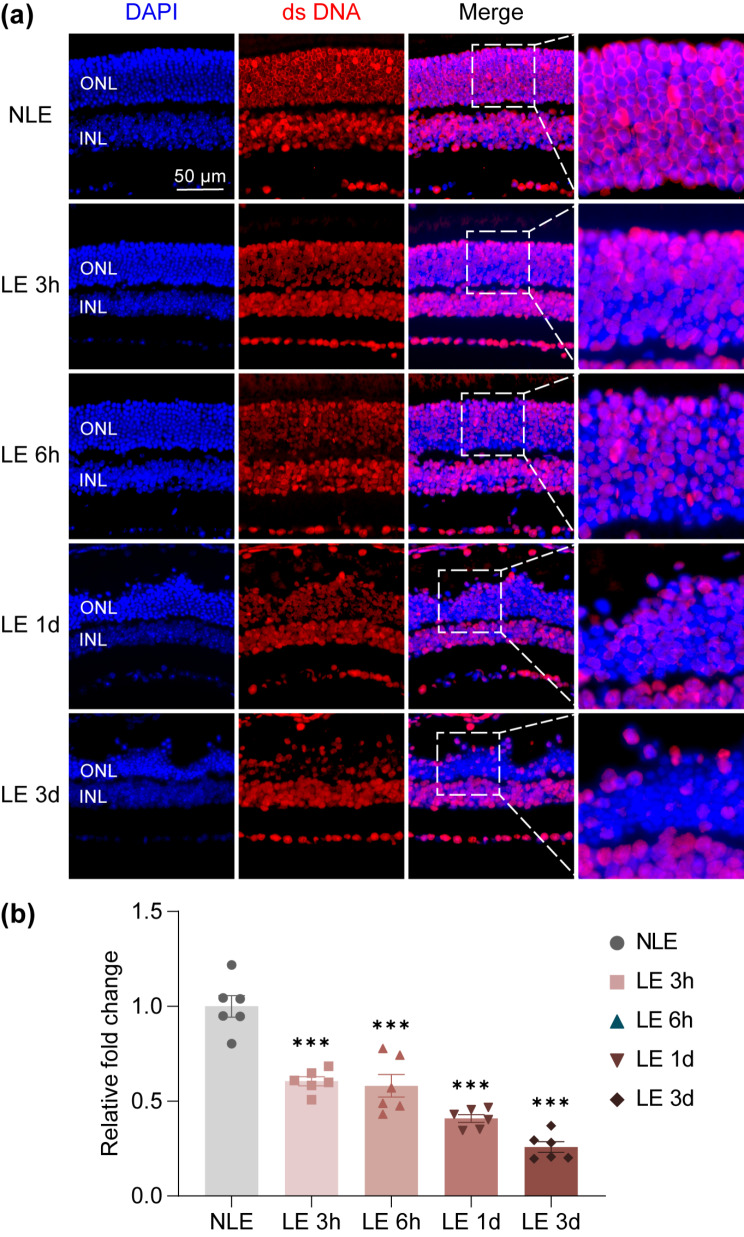
Degenerating photoreceptors are marked with diminished dsDNA in the nuclei. **(a)** Representative microscopic images of dsDNA immunopositivity (in red) and DAPI-counterstained nuclei (in blue) (*n* = 4 per group). The boxed areas within the images were magnified and displayed in the right panels. Scale bar, 50 μm. **(b)** The positivity of dsDNA in the ONL was quantified by ImageJ. Relative fold change in the positivity of dsDNA in the ONL was plotted against NLE. Data were expressed as mean ± SEM (*n* = 6 per group). ^***^Compared to NLE, *P* < 0.001. NLE, the mice without the experimental light exposure; LE, the light-exposed mice; INL, inner nuclear layer; ONL, outer nuclear layer

**Fig. 7:**
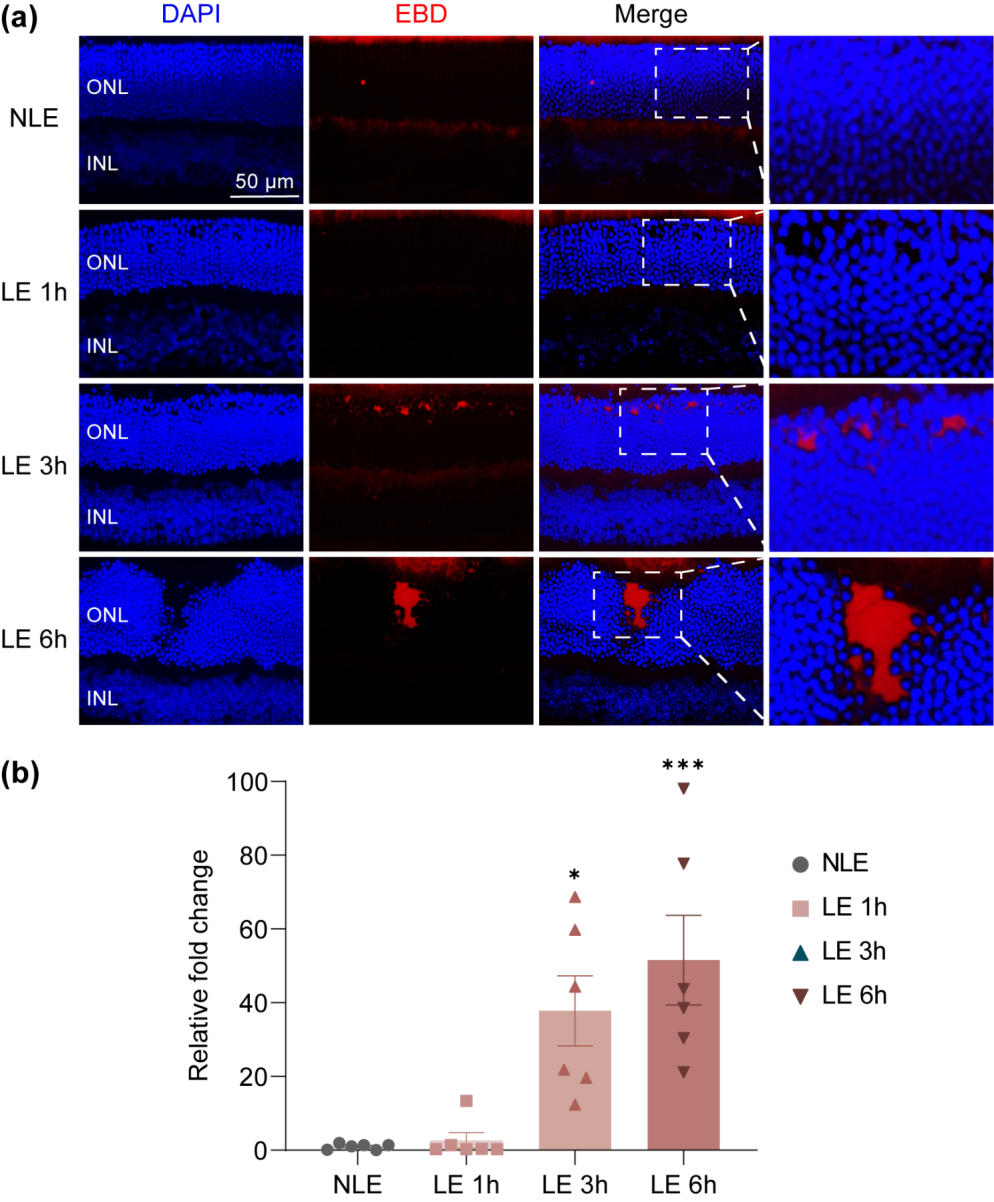
The light-exposed retinas are marked with extracellular presence of the exposed DNA. **(a)** Evans blue dye uptake assay was conducted 1 h, 3 h and 6 h after the experimental light exposure (*n* = 4 per group). Representative micrographs of DAPI fluorescence (in blue) and Evans blue autofluorescence (in red) were presented. Scale bar, 50 μm. **(b)** The area of Evans blue autofluorescence in the ONL was quantified by ImageJ. Relative fold change in the area of Evans blue autofluorescence in the ONL was plotted against NLE. Data were expressed as mean ± SEM (*n* = 6 per group). ^*^Compared to NLE, *P* < 0.05; ^***^compared to NLE, *P* < 0.001. EBD, Evans blue dye; NLE, the mice without the experimental light exposure; LE, the light-exposed mice; INL, inner nuclear layer; ONL, outer nuclear layer

Damage-associated molecular patterns (DAMPs) are important neuroinflammatory mediators linking dying neurons and activated microglia during retinal degeneration [15]. Of note, accumulating evidence has supported the important contribution of cGAS-STING pathway in sensing self-DNA, a type of DAMPs, and relaying a cascade of signaling events that eventually lead to microglial activation-mediated neuroinflammation [16, 17]. Most relevantly, remarkable activation of cGAS-STING pathway in microglia has been linked to light-induced photoreceptor degeneration [18]. We were also able to confirm STING activation in the F4/80 positive microglia/macrophages in the light-exposed retinas (Supplementary Fig. 5). Moreover, our previous RNA-sequencing analysis has demonstrated that in the light-exposed retinas, multiple pathways involved in inflammatory responses are upregulated prior to overt loss of photoreceptors [10]. Among these pathways, cytosolic DNA-sensing pathway was significantly upregulated in the light-exposed retinas as revealed by our previous gene set enrichment analysis (Fig. 5a) and heatmap visualization of the representative genes from cytosolic DNA-sensing pathway (Fig. 5b). We thus further assessed the impact of post-light damage treatment of hyperoside on the retinal expression of the genes in cytosolic DNA-sensing pathway based on our previous RNA-seq analysis. The results revealed decreased retinal expression of *Cgas*, *Ifi202b*, *Ifnb*, *Irf7*, *Ikbke* and *Sting1* in the hyperoside-treated light-exposed mice compared to the vehicle-treated light-exposed mice (Fig. 5c). Based on these results, we hypothesized that the initial photooxidative insult to photoreceptors may cause release of dsDNA from the degenerating photoreceptors. To test this hypothesis, we examined the pattern of dsDNA in the photoreceptors after the indicated treatments. As shown in Fig. 6a, in contrast to ubiquitous dsDNA signal detected in the nucleated layers of the retinas from the normal controls, overt diminishment of dsDNA positivity was only noted in the ONL but not in other nucleated retinal layers in the light-exposed retinas. Meanwhile, significant reductions in the dsDNA positivity in the ONL were observed starting from 3 h post illumination (Fig. 6b). The release of the cellular contents, e.g., genomic DNA, occurs due to disruption of the plasma membrane of the necrotic cells. Evans blue is a necrosis-avid agent that binds exposed DNA in the necrotic tissues [19]. Evans blue dye uptake assay was thus further performed to help visualize the extracellular accumulation of DNA in the light-exposed retinas. As shown in Fig. 7a, amorphous Evans blue positive signal nonoverlapping with DAPI positive nuclei was readily detected in the ONL starting from 3 h post light exposure. Quantification of the area of Evans blue autofluorescence in the ONL revealed significantly increased Evans blue dye accumulation 3 h and 6 h post light exposure (Fig. 7b). These results suggest accumulation of extracellular DNA in the ONL following light exposure. Taken together, these results support the possibility that released dsDNA accumulates in the ONL in the light-exposed retinas, which may serve as DAMPs to activate microglia during the course of photoreceptor degeneration. We thus hypothesized that hyperoside may attenuate the progression of photoreceptor degeneration in part by mitigating DNA-stimulated microglial activation.

### Hyperoside attenuates DNA-stimulated pro-inflammatory activation of microglia

**Fig. 8:**
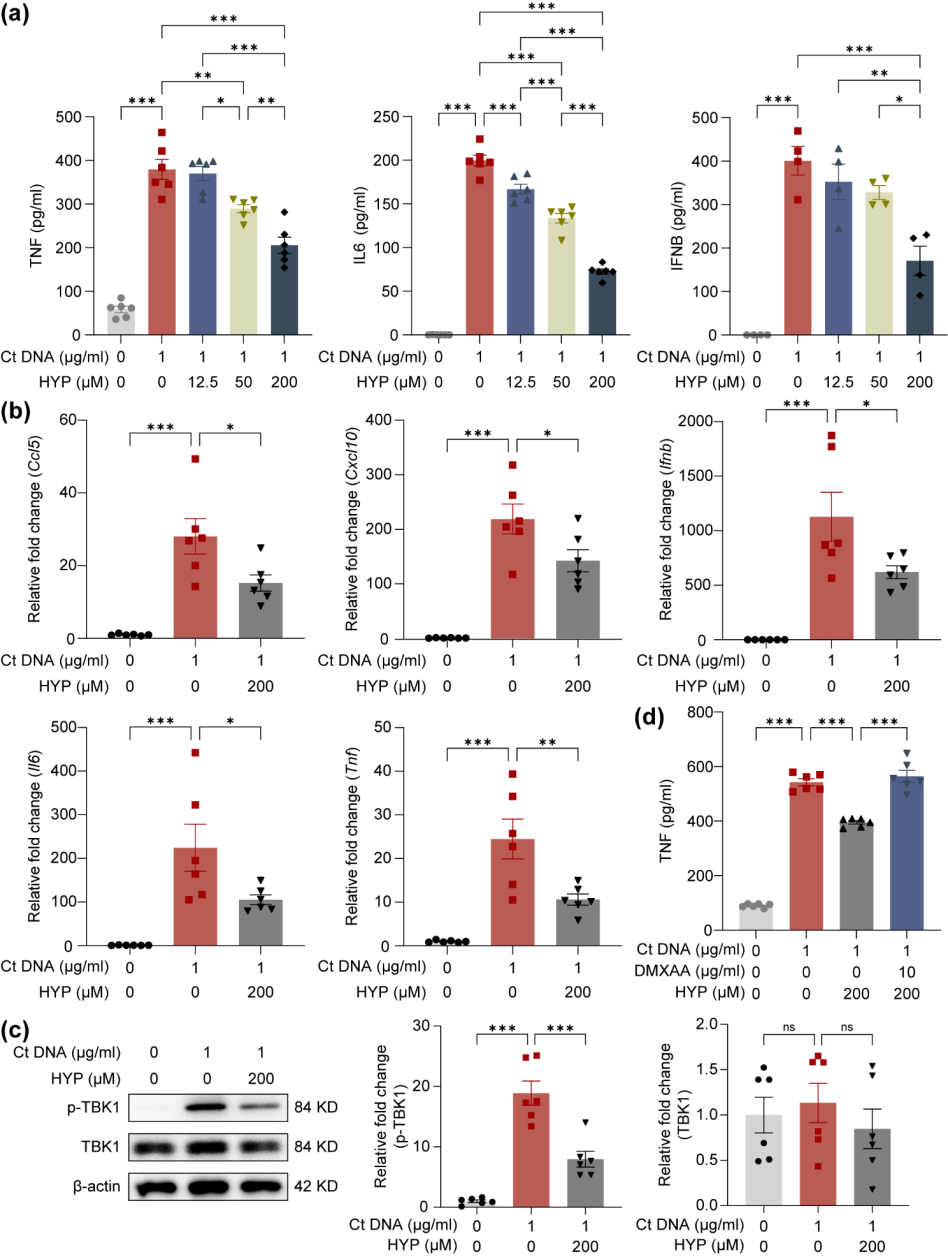
Hyperoside inhibits DNA-stimulated pro-inflammatory responses in BV-2 cells. **(a)** ELISA was performed to quantify ctDNA-stimulated production of TNF, IL6, and INFB 3 h after the indicated treatments. **(b)** Real-time qPCR analyses of the expression of *Ccl5*, *Cxcl10*, *Ifnb*, *Il6* and *Tnf* in BV-2 cells collected 3 h after the indicated treatments. Relative fold change was normalized against the vehicle-treated BV-2 cells. **(c)** Western blotting analyses of the level of p-TBK1 and TBK1 in BV-2 cells collected 3 h after the indicated treatments. β-actin was probed as the internal control. Relative fold change was normalized against vehicle-treated BV-2 cells. **(d)** ELISA was performed to quantify ctDNA-stimulated production of TNF 3 h after the indicated treatments. Data were expressed as mean ± SEM (*n* = 6 per group). ^*^P < 0.05; ^**^P < 0.01; ^***^P < 0.001; ns, not significant

To further test the hypothesis that hyperoside may suppress DNA-triggered microglia-mediated neuroinflammation, we assessed the impact of hyperoside against ctDNA-stimulated pro-inflammatory activation of BV-2 cells. As shown in Fig. 8a, ctDNA-stimulated production of TNF, IL6 and IFNB was attenuated by hyperoside in a dose-dependent manner. Additionally, given that DNA-stimulated production of the pro-inflammatory factors is regulated at the transcriptional level downstream of STING activation, the mRNA expression of *Ccl5*, *Cxcl10*, *Ifnb*, *Il6* and *Tnf*, targets of the STING pathway was further analyzed. As shown in Fig. 8b, ctDNA stimulation led to significantly upregulated expression of *Ccl5*, *Cxcl10*, *Ifnb*, *Il6* and *Tnf* in the vehicle-treated BV-2 cells. In contrast, decreased expression of *Ccl5*, *Cxcl10*, *Ifnb*, *Il6* and *Tnf* was noted in the hyperoside-treated ctDNA-stimulated BV-2 cells. Moreover, given that phosphorylation of TBK-1 signifies the activation of the STING pathway, we further analyzed the level of phosphorylated TBK-1 after the indicated treatments. The results revealed that ctDNA-stimulated phosphorylation of TBK-1 was dampened by hyperoside treatment (Fig. 8c). In addition, DMXAA, a non-cyclic dinucleotide small molecule agonist of the murine STING, abolished the suppressive effect of hyperoside on ctDNA-stimulated production of TNF (Fig. 8d). Of note, when STING, the central player of the DNA-sensing pathway was directly activated by its natural ligand 2′3′-cGAMP or DMXAA, no significant reductions in the production of TNF were observed in the presence of hyperoside treatment (Supplementary Fig. 6). Taken together, these results indicate that hyperoside is effective at counteracting the action of DNA in stimulating the pro-inflammatory activation of microglia. However, STING per se does not seem to be directly targeted by hyperoside in attenuating DNA-sensing pathway-mediated microglial activation.

#### Hyperoside directly interacts with cGAS and suppresses the activation of cGAS in microglia

**Fig. 9:**
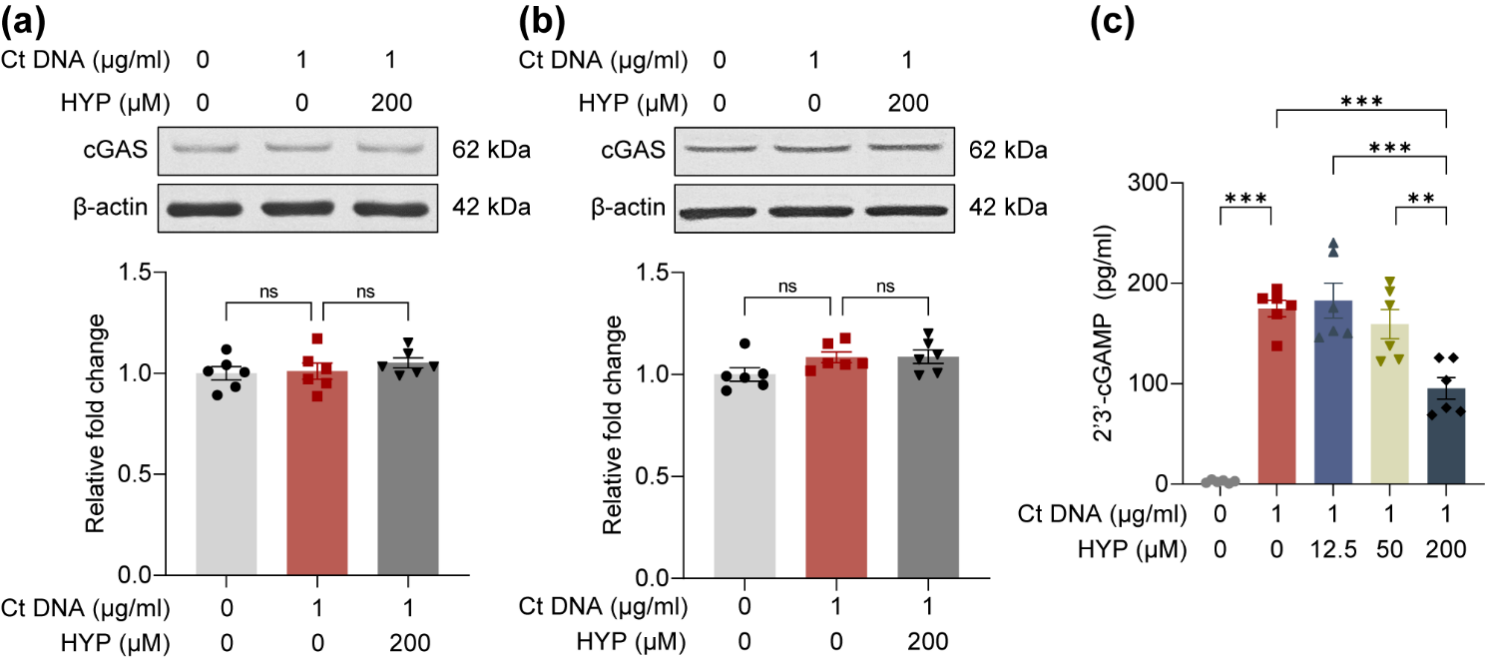
Hyperoside inhibits DNA-stimulated cGAS activation in microglia. A and B. BV-2 cells were subjected to the indicated treatments for 3 h **(a)** and 6 h **(b)**, followed by Western blotting analysis of the protein level of cGAS. β-actin served as the internal control. Relative fold change was normalized against the vehicle-treated BV-2 cells. **(c)** Three hours after the indicated treatments, BV-2 cells were collected and the level of 2′3′-cGAMP was determined by a competitive ELISA. Data were expressed as mean ± SEM (*n* = 6 per group). ^**^P < 0.01; ^***^P < 0.001; ns, no significant

**Fig. 10:**
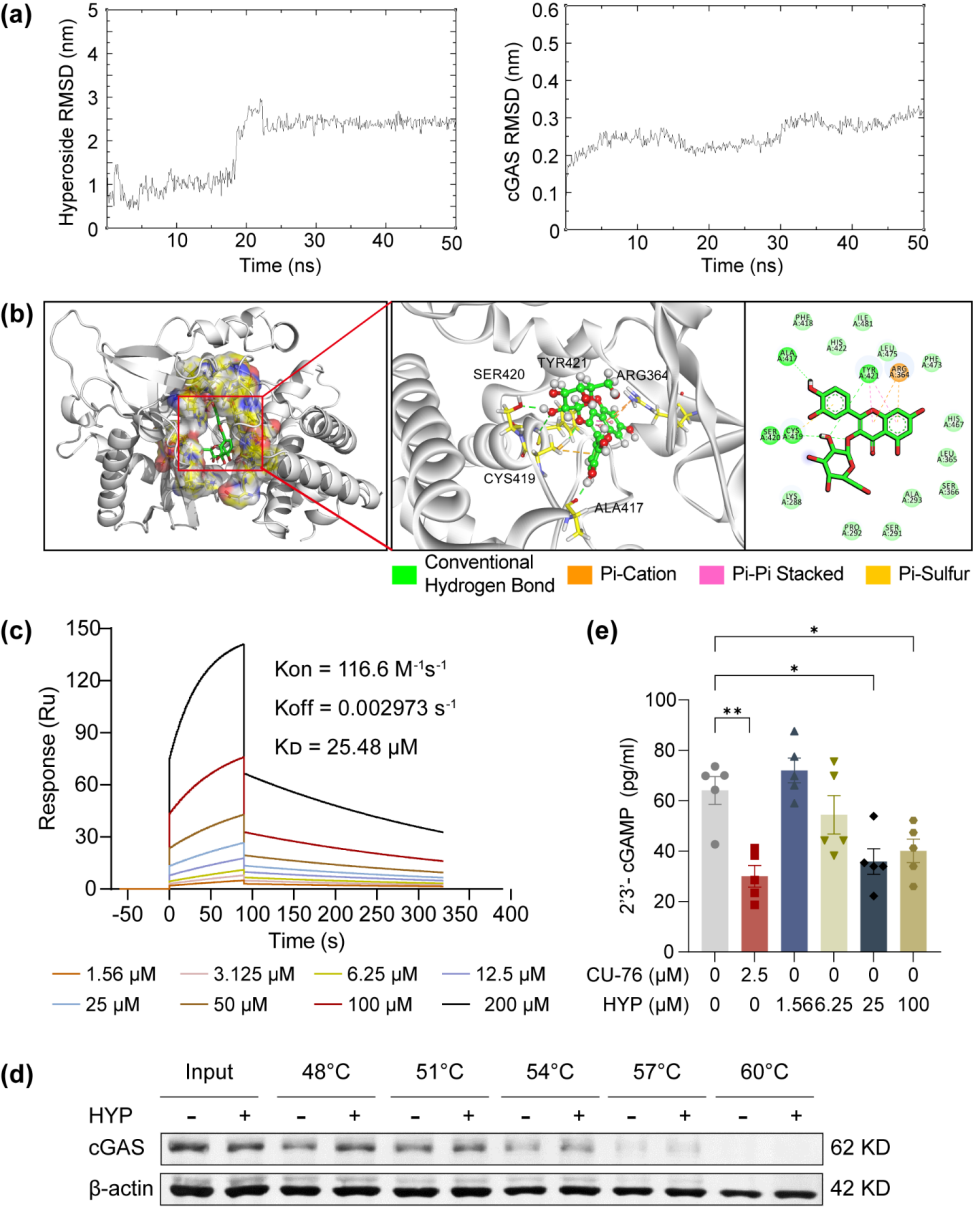
Hyperoside directly interacts with and inhibits cGAS. **(a)** MD simulations of hyperoside-mouse cGAS complex. **(b)** Binding mode of hyperoside to cGAS. **(c)** SPR analyses of the hyperoside-human cGAS interaction. **(d)** CETSA assessment of the binding of hyperoside and cGAS protein in BV-2 cells. β-actin was the internal reference. The data were representative of three independently repeated experiments. **(e)** Measurement of cGAS activity in a cell-free assay by competitive ELISA. cGAS inhibitor CU-76 was included as the positive control. Data were expressed as mean ± SEM (*n* = 6 per group). ^*^P < 0.05; ^**^P < 0.01. RMSD, root mean square deviation; ns, nanosecond

To further probe the mechanisms underlying the suppressive effects of hyperoside on DNA-sensing pathway-mediated pro-inflammatory activation of microglia, the potential impact of hyperoside on the expression and activation of cGAS was analyzed. No significant changes in the protein level of cGAS were observed in the BV-2 cells after ctDNA stimulation for 3 h (Fig. 9a) or 6 h (Fig. 9b) whether or not hyperoside was included. However, the production of 2′3′-cGAMP in the BV-2 cells was increased in response to ctDNA stimulation, whereas hyperoside treatment dose-dependently reduced the production of 2′3′-cGAMP in the ctDNA-stimulated BV-2 cells, implying that hyperoside acts on cGAS and suppresses DNA-triggered cGAS activation in the BV-2 cells (Fig. 9c). These results raised the possibility that cGAS could be directly targeted by hyperoside. To test this hypothesis, molecular docking and MD simulations were further conducted. As shown in Fig. 10a, the ligand RMSD increased to 3 nm at 20 ns and then equilibrated at approximately 25 ns. The protein RMSD was relatively stable between 30 ns to 50 ns. The binding mode of hyperoside to mouse cGAS was thus determined through MD simulations within 30 ns to 50 ns timeframe. The analysis of molecular interaction patterns revealed that four hydrogen bonds were formed between hyperoside and mouse cGAS, with which hyperoside interacting via SER420, CYS419, TYR421, and ALA417. Additionally, a pi-cation interaction with ARG364, a pi-pi interaction with TYR421, and a pi-sulfur interaction with CYS419 were noted (Fig. 10b). The binding energy was calculated to be -9.01 kcal/mol. These findings support a direct interaction of hyperoside with cGAS. Next, SPR was employed to verify the interaction and assess the binding kinetics as well as the affinity constants of hyperoside and human cGAS. The results revealed that the association rate constant (Kon), the dissociation rate constant (Koff) and the equilibrium dissociation constant (K_D_) for cGAS protein against hyperoside were 116.6 M^− 1^s^− 1^, 0.002973 s^− 1^ and 25.48 µM, respectively (Fig. 10c), indicating a relatively strong affinity between hyperoside and cGAS. CETSA was also performed to validate the interaction of hyperoside and cGAS in the BV-2 cells. As shown in Fig. 10d, in the presence of hyperoside, enhanced stability of cGAS protein was noted from 48℃ to 57℃, supporting a direct interaction between hyperoside and cGAS in microglia. Lastly, a cell-free cGAS enzymatic activity assay was adopted to validate the functional consequence of the direct interaction between hyperoside and human cGAS. Similar to the observations made in ctDNA-stimulated BV-2 cells (Fig. 9c), inclusion of hyperoside in the cell-free reaction resulted in lower level of 2′3′-cGAMP, indicating that hyperoside directly inhibits the activation of human cGAS (Fig. 10e). Collectively, these results demonstrate that hyperoside directly interacts with cGAS and suppresses the enzymatic activation of cGAS in microglia.

## Discussion

**Fig. 11:**
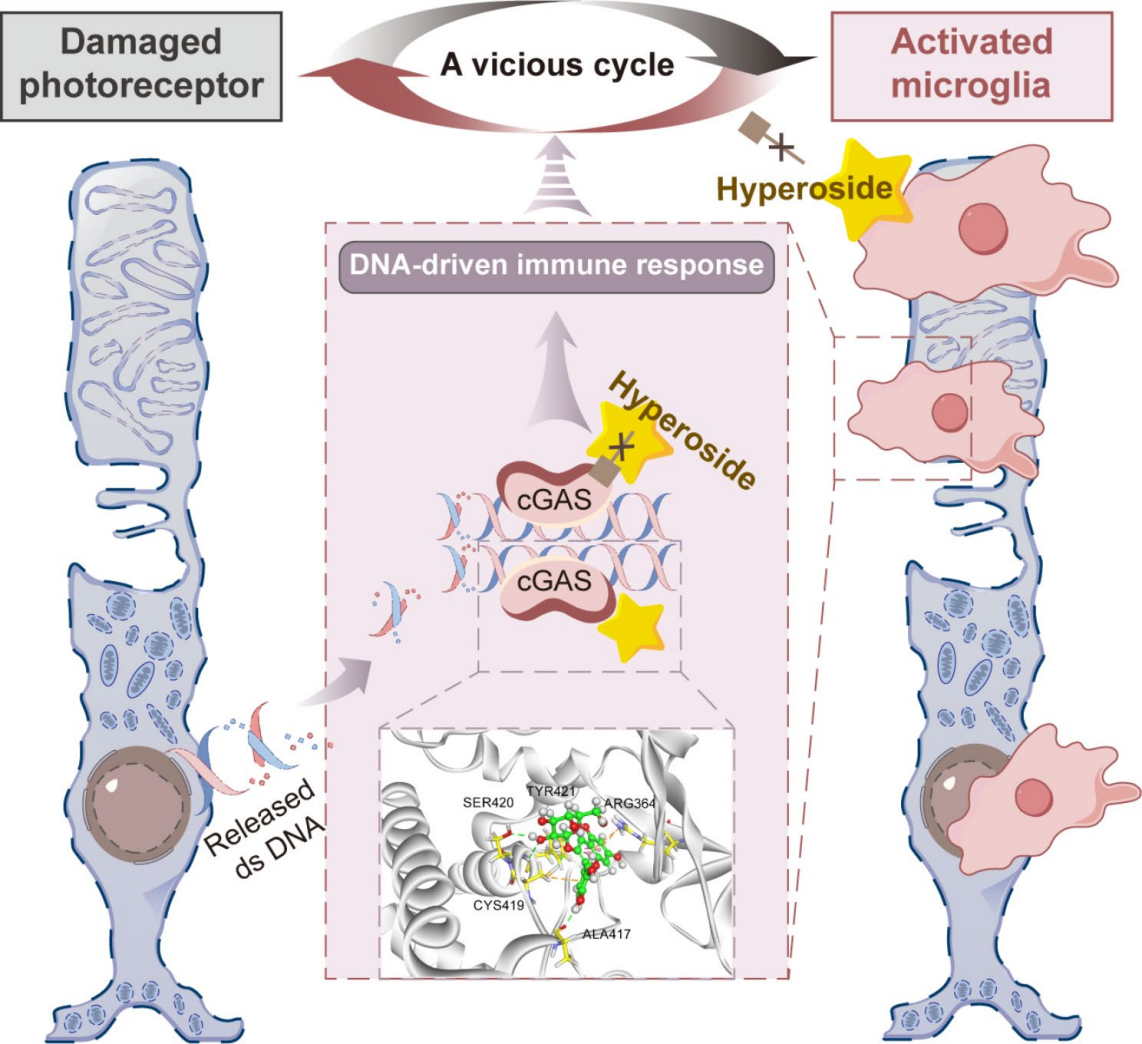
A schematic drawing: Hyperoside may target DNA sensor cGAS and break the vicious cycle of microglial activation-instigated photoreceptor loss. Hyperoside directly interacts with cGAS, suppresses cGAS activation and lowers DNA-stimulated pro-inflammatory responses in microglia, which likely contribute to its effects at mitigating microglial activation-mediated neuroinflammation and photoreceptor degeneration. Dashed line box and dashed arrow indicate findings were derived from in vitro assays

Microglial activation-mediated neuroinflammatory responses in the retina play an important role in exacerbating photoreceptor degeneration. Targeting aberrant microglial activation is therefore of significant therapeutic value in curtailing the progression of photoreceptor degeneration. The work here demonstrates that hyperoside interacts with cGAS, suppresses cGAS activation and lowers DNA-stimulated pro-inflammatory responses in microglia, which likely contributes to its effects at mitigating microglial activation-mediated neuroinflammation and photoreceptor degeneration (Fig. 11).

The major findings here relate to the pharmacological implications of hyperoside in ameliorating photoreceptor structure and retinal function through suppressing pro-inflammatory microglial activation in the pathological context of photoreceptor degeneration. Neuroinflammation has been recognized as a critical pathophysiological element in the progression of various retinal disorders, whether they are of neovascular or degenerative etiologies [20]. As the key player in maintaining the neuroimmune homeostasis, microglia have a close reciprocal relationship with retinal neurons including photoreceptors under both physiological and pathological conditions [20]. During development and under normal physiological conditions, microglia exert important homeostatic functions in regulating retinal vascularization, developmental retinal cell death and survival as well as synaptic refinement [21]. In the pathological context, however, the loss of retinal homeostasis invariably triggers pro-inflammatory microglial activation and recruitment. In the case of photoreceptor degeneration, aberrantly activated microglia migrate to the site with degenerative alterations, produce pro-inflammatory cytokines, and exacerbates the loss of photoreceptors via neurotoxic effects or phagocytosis of the viable photoreceptors [6]. In vivo findings from different photoreceptor degeneration mouse models consistently support the notion that suppression of microglial activation helps alleviate photoreceptor degeneration [22–24]. Thus, counteracting the pro-inflammatory activation of microglia and aberrant neuroinflammatory responses is regarded as a viable route to curtail the progression of photoreceptor degeneration [5]. Our previous study has demonstrated that as an antioxidant, hyperoside protects against photooxidative stress-mediated photoreceptor degeneration [10]. Other studies have indicated that as an anti-inflammatory agent, hyperoside is effective at attenuating microglial activation-mediated neuroinflammatory responses. To name a few, hyperoside suppresses LPS-stimulated pro-inflammatory activation of microglia and associated neurotoxicity in SH-SY5Y neuroblastoma cells in vitro [8]. Hyperoside also alleviates the pro-inflammatory activation of microglia in a mouse model recapitulating Parkinson’s disease [9]. However, instead of taking effect at the level of photoreceptors by counteracting oxidative stress-instigated photoreceptor damage as revealed in our earlier study [10], whether hyperoside is effective at directly suppressing photoreceptor degeneration-associated microglial activation and most importantly, whether hyperoside is able to palliate photoreceptor degeneration by curtailing the pro-inflammatory microglial activation remain to be uncovered. The in vivo findings here demonstrate that hyperoside mitigates retinal inflammation, microglial activation and photoreceptor degeneration when administered in a manner that excludes its photoreceptor protective activity. Therefore, the effects of hyperoside at suppressing the pro-inflammatory activation of microglia and neuroinflammation expand its pharmacological implications in ameliorating the outcome of photoreceptor degeneration. The evidence from the current work and our previous study [10] collectively indicate that hyperoside may exert two-fold impact on photoreceptors at different stages of the disease progression, first as an antioxidant to combat oxidative stress at the initial stage and secondly, as an anti-inflammatory agent to control aberrant microglial activation-mediated neuroinflammatory responses at the stage following photoreceptor damage. Furthermore, complex cell-cell interaction is at play in maintaining the survival and functional homeostasis of photoreceptors. For example, photoreceptors interact closely with retinal pigment epithelium (RPE) cells and Müller cells, which provide essential structural and metabolic support to the photoreceptors. During photoreceptor degeneration, RPE cells and Müller cells undergo pathological changes that have deleterious impact on the survival and function of photoreceptors. For instance, our earlier studies have shown that prior to massive loss of photoreceptors, overt oxidative stress and ultrastructural impairment of the apical microvilli are evident in RPE cells [10, 25]. Excessive oxidative damage to the RPE and structural impairment of the RPE microvilli inevitably sabotage the survival and function of the photoreceptors [26, 27]. In the meantime, during photoreceptor degeneration, Müller cells experience reactive gliosis and the retinal expression of glutamine synthetase-encoding *Glul* is significantly decreased [10]. Among the detrimental effects, Müller cells undergoing aberrant gliosis may fail to fully carry out the function of metabolizing glutamate released by the damaged photoreceptors. As a result, excessively accumulated glutamate further exacerbates photoreceptor cell death [28]. Therefore, considering the pathophysiological significance of complex cell-cell interaction in the pathogenesis of photoreceptor degeneration, it is possible that the photoreceptor protection conferred by post-light damage hyperoside treatment may also implicate the pharmacological actions of hyperoside on RPE cells and Müller cells, which remains to be elucidated in the future studies.

Moreover, the work here provides new understanding of the mechanisms underlying the effects of hyperoside at suppressing the pro-inflammatory activation of microglia. DNA is high immunogenic and can elicit strong inflammatory responses. Our in vitro findings uncover that hyperoside suppresses DNA-stimulated microglial activation in part through directly interacting with the DNA sensor cGAS and suppressing the enzymatic activity of cGAS. cGAS-STING pathway plays a critical role in detection of dsDNA and initiation of robust immune and inflammatory responses. DNA-bound cGAS catalyzes the production of 2′3′-cGAMP, a second messenger that binds and activates the adaptor protein STING. Activated STING promotes the activation of the downstream transcriptional factors such as interferon regulatory factor 3 and nuclear factor-κB that trigger the transcription of type I interferons and pro-inflammatory factors, respectively [29–31]. Although cGAS was originally recognized for sensing dsDNA and promoting STING activation in the context of foreign pathogen infection, recent studies have implicated cGAS-STING signaling as an important machinery for relaying the self DNA-stimulated inflammatory responses in a broad range of non-infectious pathological contexts including neurodegeneration. For instance, cGAS gain-of-function alone is sufficient to drive the pro-inflammatory activation of microglia and promote ageing-associated decline of the neuronal functions [16]. It has been recognized that stressed or dying photoreceptors release various kinds of DAMPs that may engage in the pro-inflammatory activation of microglia through multiple mechanisms [15]. In this study, we present new morphological evidence indicating that photoreceptor-derived self DNA is a type of DAMP released early in the photoreceptor degenerative process. This notion is supported by markedly diminished dsDNA in the photoreceptor nuclei that is followed by extracellular accumulation of Evans blue-bound exposed DNA in the ONL. Thus, the aforementioned observations collectively support the possibility that self DNA released from the stressed or dying photoreceptors may subsequently trigger cGAS activation to initiate the DNA-sensing pathway in microglia. Indeed, corroborating the evidence of STING activation in the microglia in the light-exposed retinas [18], our results also demonstrate that some of the F4/80 positive microglia/macrophages found in the outer retina are marked by phosphorylated STING. Post-light damage hyperoside treatment not only results in fewer microglia in the outer retina but also leads to lower retinal expression of the genes in DNA-sensing pathway. Furthermore, our in vitro findings support the direct impact of hyperoside on suppressing DNA-stimulated pro-inflammatory responses in microglia. Hyperoside physically interacts with cGAS, suppresses the enzymatic activity of cGAS and lowers the level of the STING pathway in DNA-stimulated BV-2 microglial cells in vitro. Future studies are worth pursuing to validate the pharmacological implications of hyperoside on photoreceptor degeneration-associated self DNA-induced microglial activation in vivo. Nevertheless, the targeted effect of hyperoside on DNA-triggered cGAS activation helps to further understand the mechanisms of actions of hyperoside in mitigating the pro-inflammatory activation of microglia. Meanwhile, self DNA is one type of DAMP released by dying cells. Other DAMPs, for instance, extracellularly released heat shock proteins and HMGB1 may also take part in activating microglia and promoting neuroinflammation [15, 32, 33]. The loss of the nuclear HMGB1 is evidenced by our previous and current study, supporting the possibility that HMGB1 is released by the dying photoreceptors [13]. Moreover, it has been shown that hyperoside attenuates exogenous HMGB1-induced inflammatory responses in endothelial cells [34]. Therefore, it is likely that other DAMPs generated by the degenerating photoreceptors, e.g., HMGB1 and heat shock proteins, may also stimulate the pro-inflammatory activation of microglia during photoreceptor degeneration. It is also possible that hyperoside restricts microglial activation stimulated by other DAMPs in the pathological context of photoreceptor degeneration. Futures studies are required to address these possibilities.

Although our results here demonstrate that hyperoside binds to cGAS and suppresses the enzyme activity of cGAS, it remains to be further studied as to how the binding of hyperoside to cGAS affects the activation of cGAS. The human cGAS protein consists of a disordered and non-conserved N-terminus (1-160) and a highly conserved C-terminus (161–522). The C-terminus contains a nucleotidyltransferase (NTase) core domain (161–330) and the male abnormal 21 (Mab21) domain (213–513). It has been shown that the DNA-sensing function of the cGAS protein is carried out by the serine (13, 37, 64, 129 and 143) residues in the N-terminus. Several conserved amino acid residues within the NTase superfamily, including G212, S213, E225, D227 and D319 are found in the C-terminal NTase domain and are required for the enzyme activity of cGAS. The conserved Zn finger motif (H390-C405) in the Mab21 domain is relevant to the specificity of cGAS toward dsDNA [35–37]. The molecular docking analysis here reveals that hyperoside interacts with cGAS via the animo acid residues distributed in the C-terminal Mab21 domain (ARG364, ALA417, CYS419, SER420, CYS419 and TYR421). Therefore, it is unlikely that hyperoside directly interferes with the DNA sensing function of cGAS. In-depth chemical biological mechanisms underlying the suppressive effects of hyperoside on the enzymatic activation of cGAS remain to be investigated in the future studies.

Lastly, in terms of the safety profiles, available findings demonstrate that the median lethal dose of orally administered hyperoside is over 5000 mg/kg in BALB/c mice and no genotoxicity has been observed [38]. Moreover, no overt toxicity has been found after 6-month oral administration of hyperoside at up to 1000 mg/kg/d, except for transient renal pathologies that recover a month after the termination of hyperoside administration [39]. Therefore, hyperoside is relatively safe and well-tolerated in vivo.

## Conclusions

In conclusion, the current study supports the notion that hyperoside is effective at palliating photoreceptor degeneration possibly by suppressing the pro-inflammatory activation of microglia. Mechanistically, hyperoside lowers the level of cytosolic DNA-sensing pathway during microglial activation, which is in part attributable to its direct interaction with cGAS and suppression of cGAS activity. Thus, the novel understanding of the pharmacological potential of hyperoside in controlling the neuroinflammatory responses in the retina may pave the way for developing effective treatment that curtails the progression of photoreceptor degeneration.

### Electronic supplementary material

Below is the link to the electronic supplementary material.

Supplementary Material 1

## Data Availability

Data will be made available by the corresponding author upon reasonable request.

## Abbreviations

2-APB: 2-Aminoethyl diphenylborinate
CCL2: Chemokine ligand 2
CETSA: Cellular thermal shift assay
cGAMP: Cyclic guanosine monophosphate–adenosine monophosphate
cGAS: Cyclic GMP-AMP synthase
ctDNA: Calf thymus DNA
DAMPs: Damage-associated molecular patterns
DAPI: 4-6-Diamidino-2-phenylindole
DMEM: Dulbecco’s modified eagle’s medium
DMSO: Dimethyl sulfoxide
DMXAA: 5,6-Dimethyl-9-oxo-9 H-xanthene-4-acetic acid
dsDNA: Double stranded DNA
ELISA: Enzyme-linked immunosorbent assay
ERG: Electroretinography
FBS: Fetal bovine serum
HE: Hematoxylin and eosin
HMGB1: High mobility group box 1
Iba-1: Ionized calcium binding adaptor molecule 1
IFNB: Interferon beta
IHC: Histological examination and immunohistochemistry
IL6: Interleukin 6
INL: Inner nuclear layer
IS: Inner segment
K_D_: Equilibrium dissociation constant
K_off_: Dissociation rate constant
K_on_: Association rate constant
LPS: Lipopolysaccharides
MD: Molecular dynamics
OCT: Optical coherence tomography
ONH: Optic nerve head
ONL: Outer nuclear layer
OPL: Outer plexiform layer
OS: Outer segment
PDB: Protein data bank
qPCR: Quantitative PCR
RMSD: Root mean square deviation
SEM: Standard error of the mean
SPR: Surface plasmon resonance
STING: Stimulator of interferon genes
TBK1: Tank binding kinase 1
TNF: Tumor necrosis factor
