# Supplementary material for: Hyperoside mitigates photoreceptor degeneration in part by targeting cGAS and suppressing DNA-induced microglial activation
Source: Acta Neuropathol Commun. 2024 May 16;12:76. doi: 10.1186/s40478-024-01793-0 (PMC11097432; doi:10.1186/s40478-024-01793-0)
Supplement: Supplementary file 1 — Supplementary Material 1 [file 40478_2024_1793_MOESM1_ESM.docx]

**Supplementary Figure 1**


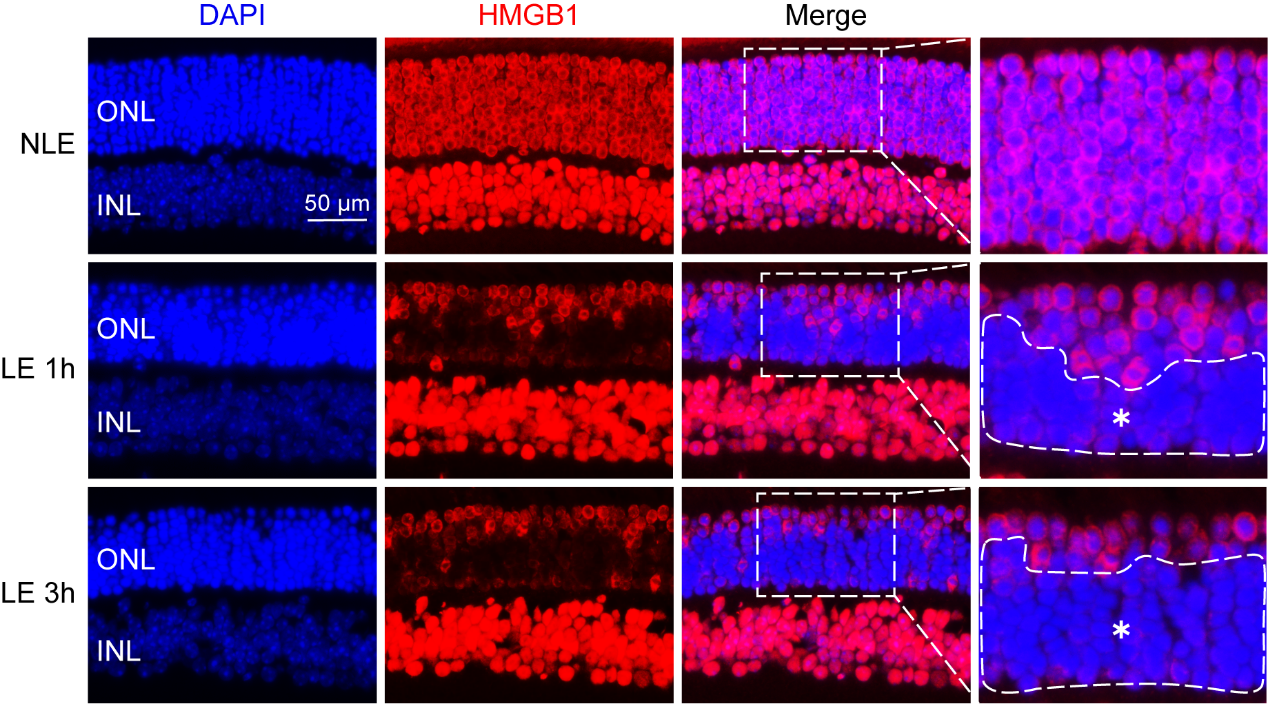


**Supplementary Figure 1. Light-induced photoreceptor damage is marked by diminishment in the nuclear HMGB1.** IHC was performed to examine the immunopositivity of HMGB1 in the retinas. The nuclei were visualized by DAPI counterstaining. Scale bar: 50 μm. Asterisks in the boxed area mark remarkable diminishment of the nuclear HMGB1. NLE, the mice without the experimental light exposure; LE, the mice exposed to the experimental light.

**Supplementary Figure 2.**


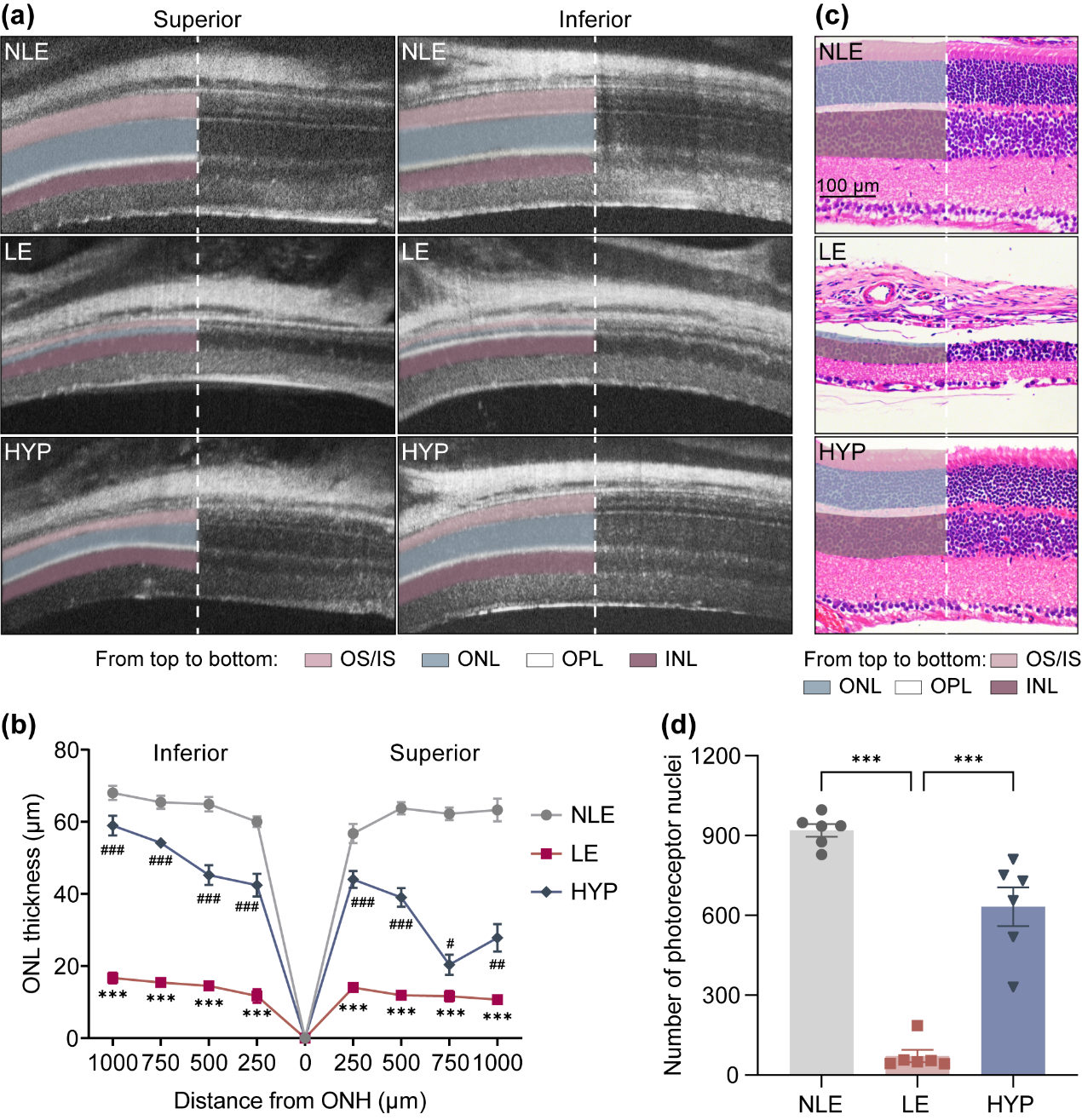


**Supplementary Figure 2. Post-light damage treatment of hyperoside confers partial protection to the photoreceptor structure.** Hyperoside was administered starting from 3 h post illumination and carried out twice a day for 7 d. **(a)** Representative OCT scans of the superior and inferior retinas. **(b)** The ONL thickness measured at 250, 500, 750, and 1000 μm away from the ONH. **(c)** Representative retinal images from the HE-stained eye sections. Scale bar, 100 μm. **(d)** The number of photoreceptor nuclei in the ONL was counted at 500 μm from ONH in the superior and inferior retinas. Data were expressed as mean ± SEM (n = 6 per group). ^***^Compared to NLE, P < 0.001; ^#^compared to LE, P<0.05, ^##^compared to LE, P<0.01, ^###^compared to LE, P<0.001. NLE, the vehicle-treated mice without light exposure; LE, the light-exposed mice treated with vehicle; HYP, the light-exposed mice treated with hyperoside; INL, inner nuclear layer; IS, inner segment; ONH, optic nerve head; ONL, outer nuclear layer; OPL, outer plexiform layer; OS, outer segment.

**Supplementary Figure 3**


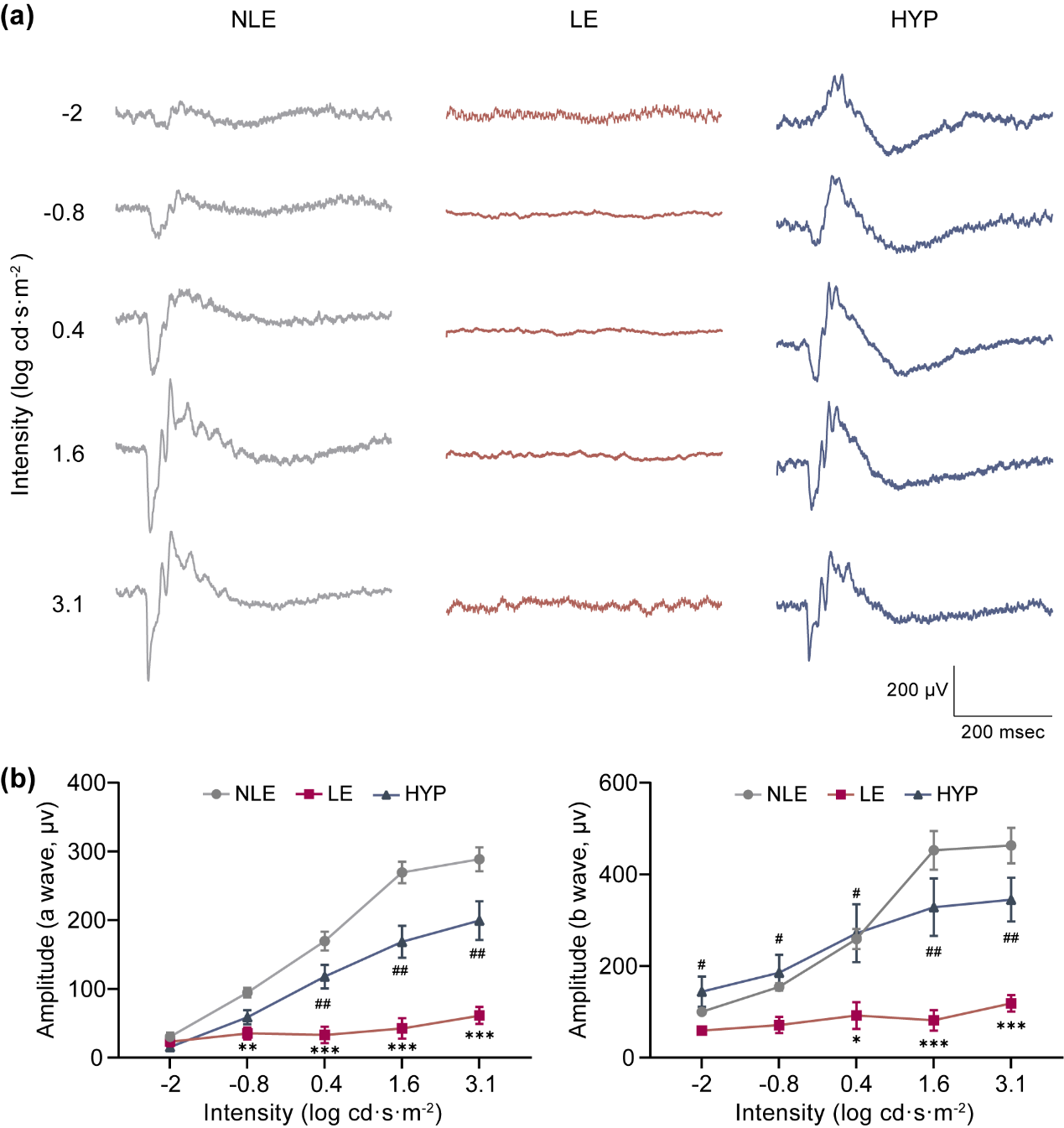


**Supplementary Figure 3. Post-light damage treatment of hyperoside maintains the retinal function.** ERG was recorded after the indicated treatments. **(a)** Representative scotopic ERG waves. **(b)** Amplitudes of a wave and b wave were plotted. Data were presented as mean ± SEM (n = 6 per group). ^*^Compared to NLE, P < 0.05; ^**^compared to NLE, P < 0.01; ^***^compared to NLE, P < 0.001; ^#^compared to LE, P < 0.05; ^##^compared to LE, P < 0.01. NLE, the vehicle-treated mice without the experimental light exposure; LE, the vehicle-treated light-exposed mice; HYP, the hyperoside-treated light-exposed mice.

**Supplementary Figure 4**


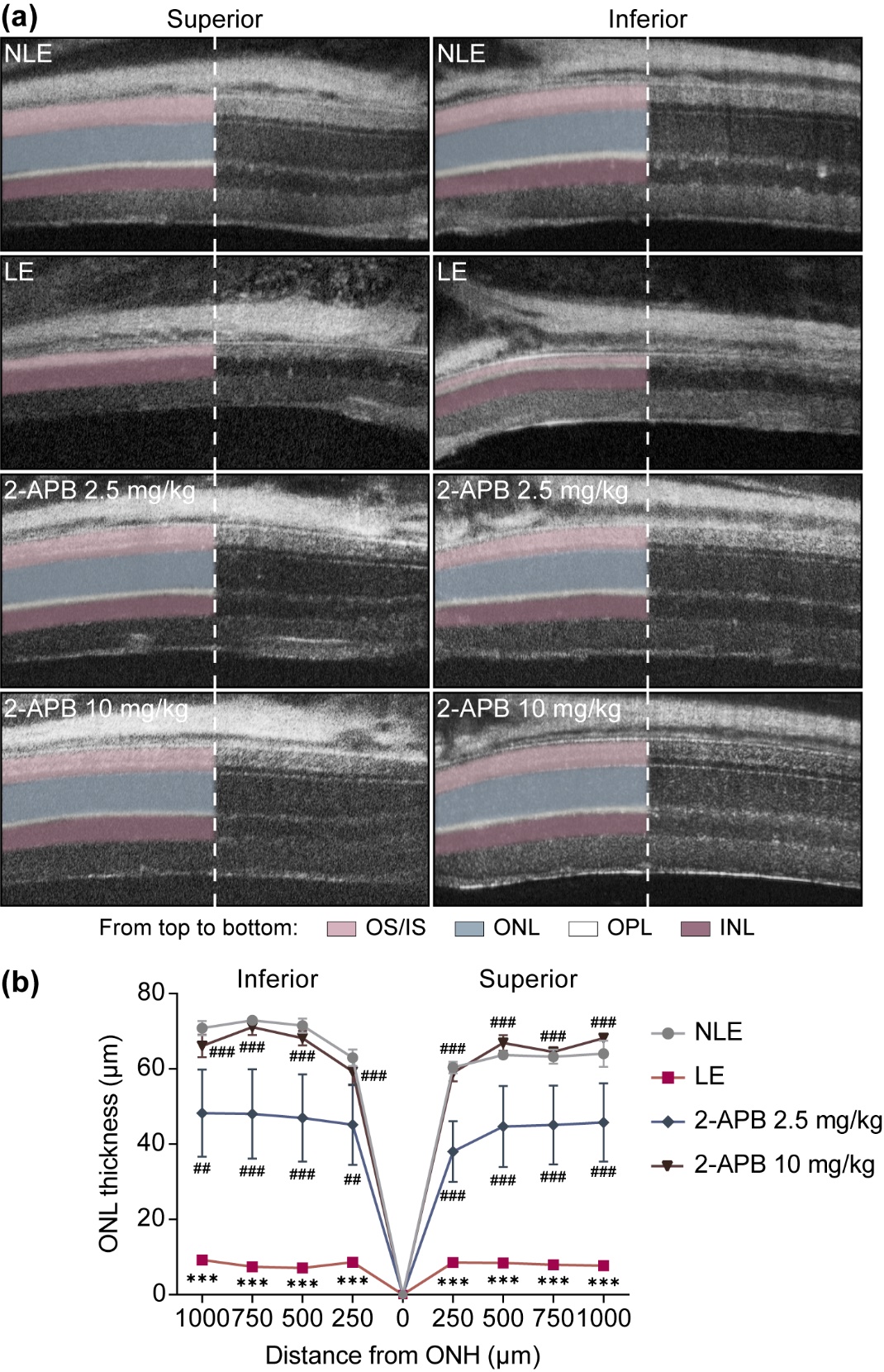


**Supplementary Figure 4. Pre-treatment of 2-APB prevents light-induced photoreceptor structural impairment.** 2-APB was administered 30 min prior to illumination. The retinal structure was assessed by OCT imaging 7 d later. **(a)** Representative OCT scans of the superior and inferior retinas. **(b)** The ONL thickness measured at 250, 500, 750, and 1000 μm away from the ONH. Data were expressed as mean ± SEM (n = 6 per group). ^***^Compared to NLE, P < 0.001; ^##^compared to LE, P<0.01, ^###^compared to LE, P<0.001. NLE, the vehicle-treated mice without light exposure; LE, the light-exposed mice treated with vehicle; 2-APB, the light-exposed mice treated with 2-APB; INL, inner nuclear layer; IS, inner segment; ONH, optic nerve head; ONL, outer nuclear layer; OPL, outer plexiform layer; OS, outer segment.

**Supplementary Figure 5**


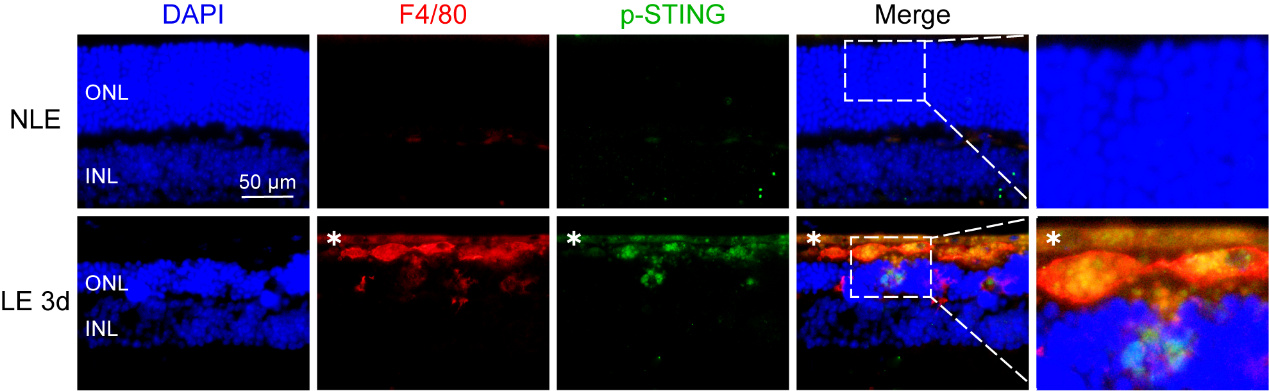


**Supplementary Figure 5. STING is activated in microglia/macrophages in the light-exposed retinas.** F4/80 positive microglia/macrophages (in red), phosphorylated STING (p-STING) (in green), and DAPI counterstaining (in blue) of the nuclei were revealed by IHC examinations. Scale bar: 50 μm. White asterisks indicate nonspecific background. NLE, the mice unexposed to the experimental light exposure; LE, the light-exposed mice; INL, inner nuclear layer; ONL, outer nuclear layer.

**Supplementary Figure 6**


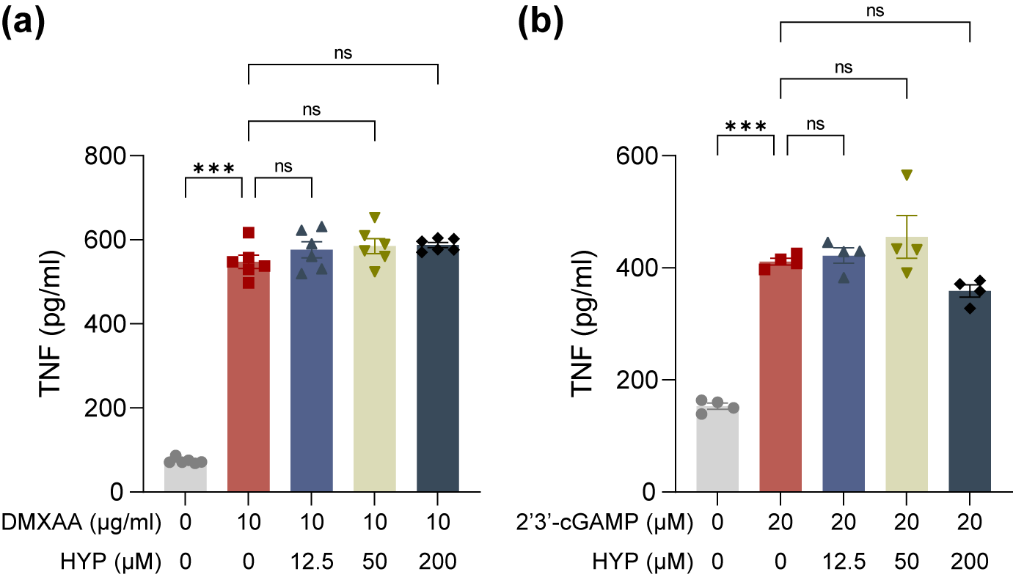


**Supplementary Figure 6. Hyperoside exerts no effects on STING agonists-triggered pro-inflammatory responses in BV-2 cells. (a)** Measurement of TNF production in DMXAA-stimulated cells in the absence or presence of hyperoside treatment. **(b)** Measurement of TNF production in 2′3′-cGAMP-stimulated cells in the absence or presence of hyperoside treatment. Data were expressed as mean ± SEM (n = 6 per group). ^***^P < 0.001; ns, no significant.
